# CDI/CDS system-encoding genes of *Burkholderia thailandensis* are located in a mobile genetic element that defines a new class of transposon

**DOI:** 10.1371/journal.pgen.1007883

**Published:** 2019-01-07

**Authors:** Angelica B. Ocasio, Peggy A. Cotter

**Affiliations:** Department of Microbiology and Immunology, University of North Carolina-Chapel Hill, NC, United States of America; Swiss Federal Institute of Technology Lausanne (EPFL), SWITZERLAND

## Abstract

Intercellular communication and self-recognition are critical for coordinating cooperative and competitive behaviors during sociomicrobiological community development. Contact-dependent growth inhibition (CDI) proteins are polymorphic toxin delivery systems that inhibit the growth of non-self neighboring bacteria that lack the appropriate immunity protein. In *Burkholderia thailandensis*, CDI system proteins (encoded by *bcpAIOB* genes) also induce cooperative behaviors among sibling (self) cells, a phenomenon called contact-dependent signaling (CDS). Here we describe a mobile genetic element (MGE) that carries the *bcpAIOB* genes in *B*. *thailandensis* E264. It is a ~210 kb composite transposon with insertion sequence (IS) elements at each end. Although the ISs are most similar to IS*2* of *Escherichia coli*, the transposase-dependent intermediate molecule displays characteristics more similar to those of the IS*26* translocatable unit (TU). A reaction requiring only the “left” IS-encoded transposase results in formation of an extrachromosomal circular dsDNA intermediate (“the megacircle”) composed of the left IS and the sequences intervening between the ISs. Insertion of the megacircle into the chromosome occurs next to a pre-existing copy of an IS*2*-like element, recreating a functional composite transposon. We found that BcpA activity is required for megacircle formation, and in turn, megacircle formation is required for CDS phenotypes. Our data support a model in which the *bcpAIOB* genes function as both helping and harming greenbeard genes, simultaneously enhancing the fitness of self bacteria that possess the same allele plus tightly linked genes that mediate cooperative behaviors, and killing non-self bacteria that do not possess the same *bcpAIOB* allele. Mobility of the megacircle between cells could allow bacteria invading a community to be converted to self, and would facilitate propagation of the *bcpAIOB* genes in the event that the invading strain is capable of overtaking the resident community.

## Author summary

As social organisms, bacteria have evolved multiple ways to communicate and interact with their neighbors. Some of these interactions can be beneficial or harmful to certain members of the community, and others involve sharing of genetic material capable of transforming the recipient cell. In this study, we provide evidence for a mobile genetic element that carries the genes encoding proteins involved in bacterial killing (contact-dependent inhibition, CDI) or cooperation (contact-dependent signaling, CDS) within microbial communities. Our findings suggest the element mobilizes with a copy-out-paste-in mechanism that requires formation of a large circular DNA molecule we call “the megacircle”. We also show that production of the megacircle requires a functional CDI/CDS system and that synthesis of the megacircle is necessary for cooperation-associated phenotypes. We hypothesize that acquisition of the megacircle provides a means to transform a target cell that does not produce the same CDI/CDS system into one that is immune to inhibition via CDI, and that can participate in the cooperative behaviors of the community.

## Introduction

Bacteria typically live in complex, dynamic, polymicrobial communities, and hence have evolved mechanisms to cooperate and compete with neighboring microbes to ensure efficient resource utilization and community survival [1–4]. Competitive interactions within communities are especially influential because of their contributions to evolution and genetic diversification [5]. One type of interbacterial competitive interaction is mediated by contact-dependent growth inhibition (CDI) systems [6]. CDI systems are composed of two-partner secretion (TPS) pathway proteins and are widespread among Gram-negative bacteria [6–8]. They fall into two main classes, *Burkholderia-*type, which are encoded by *bcpAIOB* genes, and *Escherichia coli*-type, which are encoded by *cdiBAI* genes [8]. The *bcpB/cdiB* genes encode the TpsB family outer membrane channel proteins, BcpB or CdiB, that translocate the large TpsA family exoproteins, BcpA or CdiA, to the cell surface. Delivery of the C-terminal toxin domain of BcpA or CdiA to a neighboring bacterium upon cell-cell contact results in growth inhibition or death, unless the recipient cell produces the correct BcpI or CdiI immunity protein [8–10].

A hallmark of CDI systems is their polymorphic nature. The N-terminal ~2,800 amino acids (aa) of BcpA/CdiA proteins are highly conserved, while the C-terminal ~300 aa (referred to as BcpA-CT or CdiA-CT) are variable. Distinct motifs, Nx(E/Q)LYN in BcpA and VENN in CdiA, separate the conserved and variable regions. The aa sequence of the BcpI and CdiI proteins are also polymorphic, and co-vary with BcpA-CT and CdiA-CT, respectively. Several BcpA-CT and CdiA-CT have demonstrated DNase or tRNase activity [9–12], and BcpI and CdiI proteins protect from such activity by binding to cognate (encoded by the same allele) but not non-cognate (encoded by a different allele) BcpA-CT or CdiA-CT [8–10]. Because CDI systems distinguish “self” from “non-self” neighbors based on a single allele, they have been implicated in kind selection, also known as “the greenbeard effect”. Kind selection provides a mechanism for indirect fitness in which a gene encoding a cooperative or altruistic behavior, or one that is closely linked, encodes a recognizable trait (e.g., a green beard), allowing individuals carrying the same allele to be recognized directly, irrespective of genealogy [13–16]. *bcpAIOB* and *cdiBAI* genes have been hypothesized to function as “harming greenbeard genes”; they encode proteins that cause harm to individuals that do not possess the same allele, thereby providing a fitness advantage to individuals that do possess the same allele.

Several recent studies have investigated the mechanism by which BcpA-CT and CdiA-CT are delivered to the cytoplasm of target bacteria. In *E*. *coli*, a region near the center of the CdiA protein binds to either BamA or a hetero-oligomeric complex of OmpF and OmpC, depending on the specific *cdiA* allele, to mediate delivery of CdiA-CT into the periplasm, and then the N-terminal half of CdiA-CT mediates translocation across the cytoplasmic membrane by interacting with a specific integral cytoplasmic membrane protein, also in an allele-specific manner [17–20]. For some CdiA proteins, another layer of specificity exists in that catalytic (toxic) activity requires an accessory protein produced by the target cell [21–23]. These data demonstrate that specificity extends beyond the interaction between BcpA-CT/CdiA-CT and BcpI/CdiI, and indicate that the only cells that are susceptible to CDI may be those that are so closely related that they also contain the same *cdiBAI* or *bcpAIOB* allele [14,24]. These observations raise the question of whether interbacterial competition is the true, or main, function of *bcpAIOB-* and *cdiBAI*-encoded proteins in nature.

We have shown that in addition to mediating competitive interactions, CDI system proteins in *Burkholderia thailandensis* E264 (*Bt*E264) induce cooperative behaviors, such as biofilm formation [25]. Other phenotypes that require BcpA catalytic activity and BcpI (to, at least, prevent BcpA-CT-mediated toxicity) include production of polysaccharides that bind Congo Red (CR) dye, production of a yellow-gold color to colony biofilms that we postulate reflects production of an unidentified pigment, and aggregation of cells at the air-liquid interphase when grown in defined medium [26]. We refer to BcpA-dependent changes in gene expression resulting in biofilm formation, CR binding, pigment production, and perhaps other community behaviors as contact-dependent signaling (CDS, [26]). We hypothesize that the BcpA-CT that is delivered to a recipient cell forms a complex with BcpI, and that this complex somehow causes a change in gene expression, perhaps by binding to regulatory sites in the chromosome, catalyzing limited nicking of the chromosome, or changing the concentration of second messengers such as c-di-GMP or cAMP [14,26]. We propose that by inducing cooperative behaviors among bacteria that possess the same allele, *bcpAIOB* genes function as “helping greenbeard genes”.

A characteristic of greenbeard genes is linkage disequilibrium between the gene encoding the recognizable trait and the gene(s) encoding cooperative or altruistic behavior [15,16]. In bacteria, genes located on the same mobile genetic element (MGE), such as a bacteriophage, a plasmid, or a genomic island, display features of linkage disequilibrium in that they move together via transduction or transformation from one cell to another. Here, we report the serendipitous discovery of a genetic element containing the *bcpAIOB* genes in *Bt*E264, and provide evidence that this element is currently mobile and defines a new class of transposon.

## Results

### The *bcpAIOB* locus of *Bt*E264 is located in a 210 kb multicopy DNA segment that is flanked by IS elements

**Fig 1:**
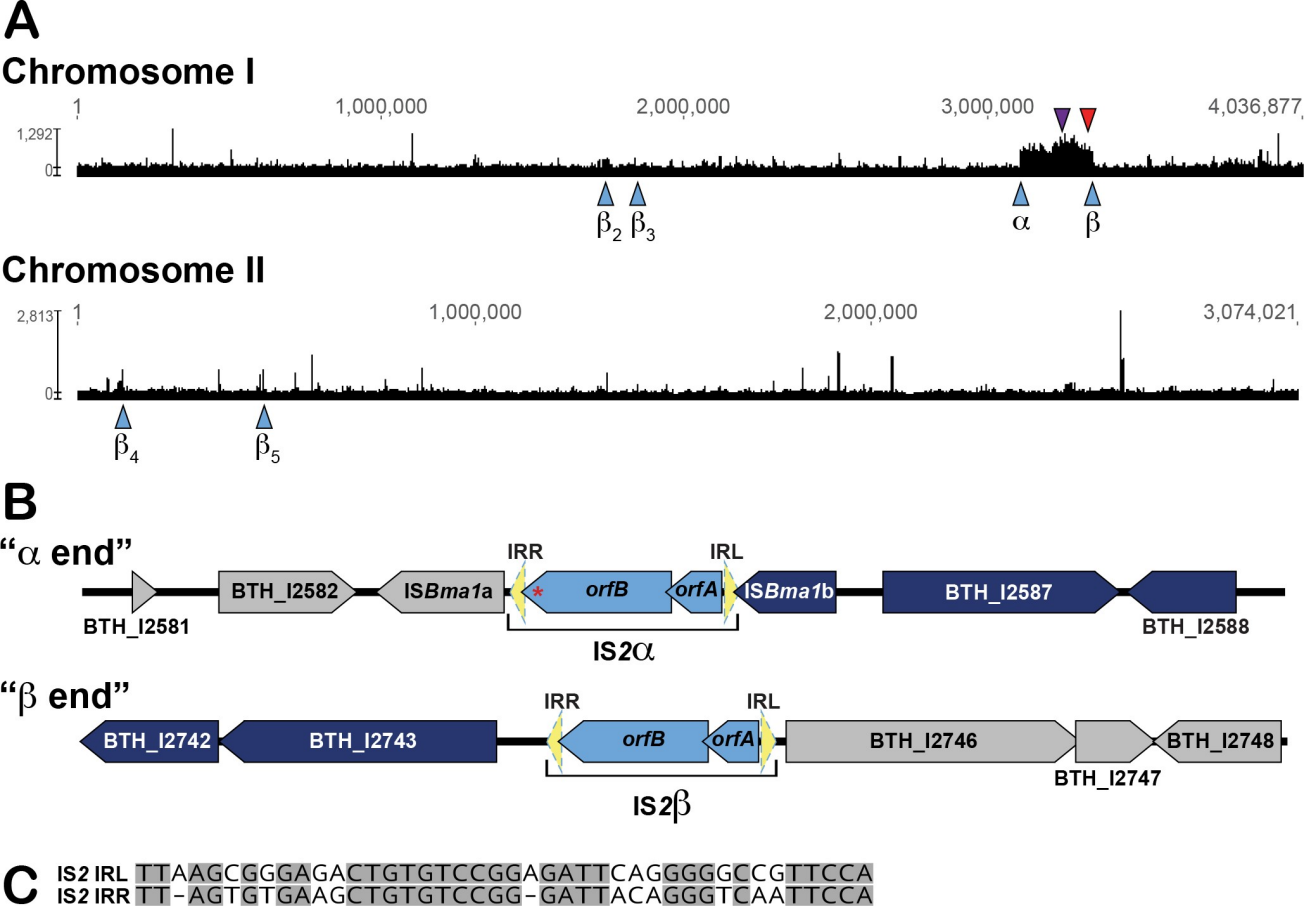
Region of increased coverage observed in WT *Bt*E264 re-sequencing analyses. **(A**) Graphical representation of genomic coverage after mapping NGS reads to chromosomes I and II of the *Bt*E264 reference genome. Left end of the region with increased coverage in chromosome I is marked as α, and the right end as β. Location of all six IS*2*-like elements (light blue), *csu* operon (purple), and *bcpAIOB* operon (red) are indicated with arrowheads. (**B**) Diagram depicting a closer view of the beginning and end of the sequence with increased coverage. Elongated pentagons represent genes outside (light grey) or inside (dark blue) of the high-coverage region. IS*2*-like elements are composed of *orfAB* (light blue) flanked by two inverted repeats (yellow). The single nucleotide variation between IS*2*α and IS*2*β is shown as a red asterisk. (**C**) Alignment using ClustalW of the IS*2* right and left imperfect repeats. Shaded nucleotides are conserved in both repeats.

Using next generation sequencing (NGS) technology, we performed a re-sequencing analysis of the complete wild-type (WT) *Bt*E264 genome. The analysis yielded a coverage graph showing how many times non-gap characters aligned to each nucleotide in the reference sequence (Fig 1A). We observed a region in chromosome I for which a high number of sequencing reads were aligned. This region has a mean coverage of 605, while the rest of chromosome I and chromosome II had mean coverages of 227.3 and 218.3, respectively. The high-coverage region spans 209,962 bps and includes 161 predicted open reading frames (S1 Table), including the *bcpAIOB* operon encoding the BcpAIOB CDI/CDS system. Genes annotated as insertion sequence (IS) elements are present at both ends of the region with high coverage. The “α end” (Fig 1B) contains two distinct genes annotated as IS*Bma*1 transposable elements and are predicted to encode IS*L3* family transposases. Although both genes were given the same annotation, they are dissimilar. To distinguish them, we have added a letter to the gene name and refer to them as IS*Bma*1a (BTH_I2583) and IS*Bma*1b (BTH_I2586). BTH_I2584 and BTH_I2585 (*orfB*_α_ and *orfA*_α_) are overlapping genes predicted to encode a single transposase with similarity to the one required for transposition of IS*2* in *E*. *coli* (S1 Fig, [27–32]). The “β end” of the 210 kb region also contains overlapping genes, BTH_I2744 and BTH_I2745. BTH_I2745 is identical to BTH_I2585 (*orfA*_*α*_), and BTH_I2744 is identical to BTH_I2584 (*orfB*_*α*_) except for a single silent nucleotide variation near the 3’ end. IS*2* elements belong to the large IS*3* family of IS elements. A common feature of this family is a programmed -1 translational frameshift that results in production of the OrfAB fusion protein, which is the functional transposase that mediates mobilization of the element [32,33]. Similar to the IS*2* from *E*. *coli*, the *Bt*E264 IS*2*-like transposase-encoding genes are flanked by imperfect inverted repeats, with the left (IRL) and right (IRR) repeat located 5’ to *orfA* and 3’ to *orfB*, respectively (Fig 1B and 1C; [32]). There are four additional IS*2*-like elements in the *Bt*E264 genome, all of which are identical to IS*2*β (Fig 1A and S2 Fig). All six IS*2*-like elements in *Bt*E264 are flanked by 5 bp target repeats, likely generated during integration of the element, a characteristic also observed for *E*. *coli* IS*2*. We did not observe increased coverage of sequences flanking any of the IS elements in our NGS analyses other than IS*2*α and IS*2*β.

### The *bcpAIOB*-containing chromosome region forms an IS*2*-dependent megacircle

Increased coverage of a contiguous region in NGS analyses could result from a duplication of the sequence compared to the reference genome. However, we have constructed several strains that contain mutations within the ~210 kb region, such as disruption of the *csu* Type-4 pilus-encoding operon (BTH_I2681-I2674; [26]) and deletions within the *bcpAIOB* locus [8]. In all cases, PCR analyses indicate that the mutations were constructed as intended, insertion borders are as expected or deleted DNA is undetected, and the mutant strains are stable and display reproducible phenotypes. These results are inconsistent with the presence of multiple, tandem copies of the ~210 kb region in the chromosome.

**Fig 2:**
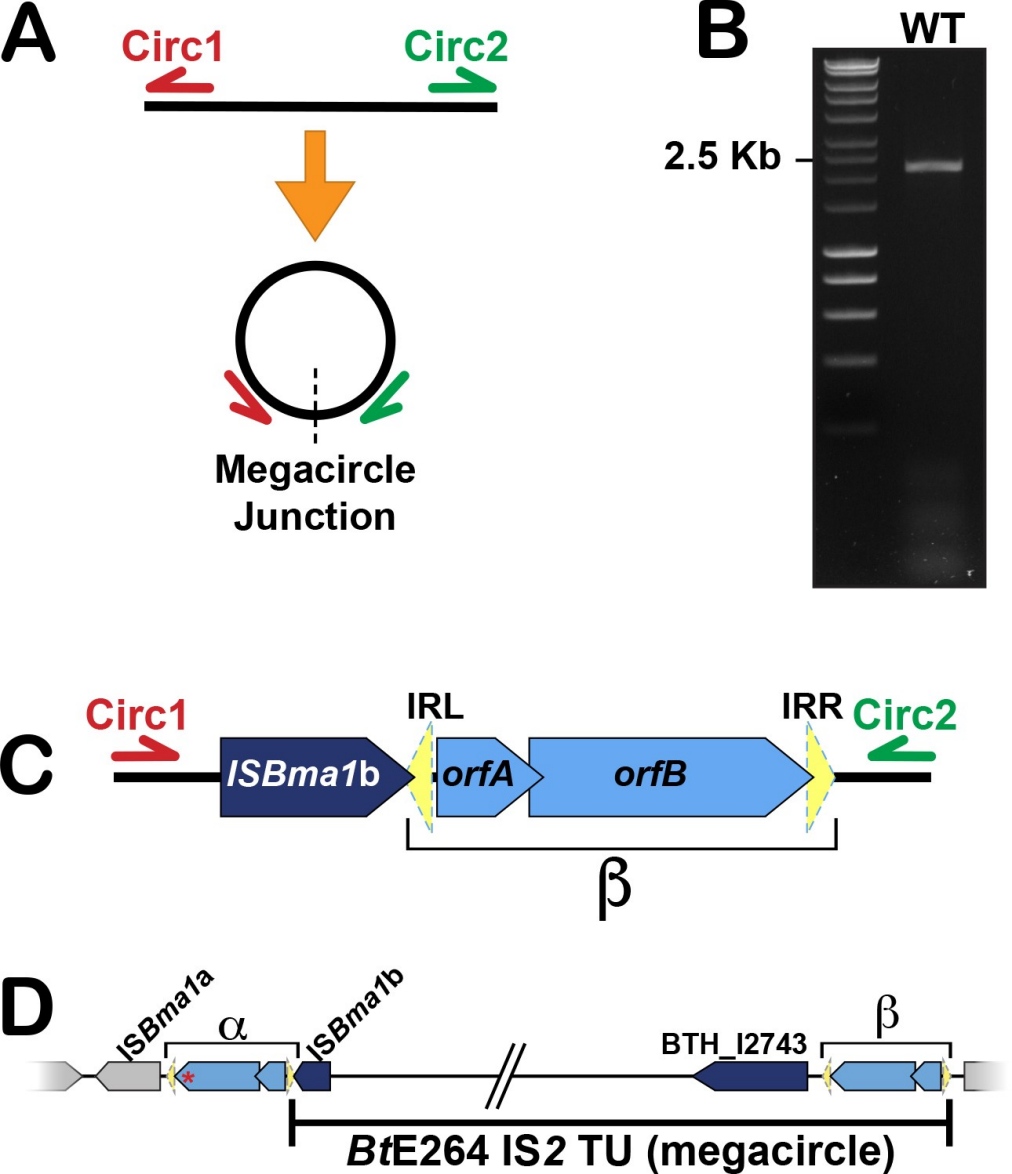
The *bcpAIOB* Locus is part of a composite transposon that forms an extrachromosomal megacircle. (**A**) Schematic representation of binding sites for primers Circ1 and Circ2 in the chromosome, as well as the IS*2*-like megacircle. (**B**) Agarose gel electrophoresis analyses show detection of the megacircle junction in WT *Bt*E264 by PCR. (**C**) Diagram of the IS*2*-like megacircle junction based on sequencing data. (**D**) Diagram of the putative IS*2*-like composite transposon. The predicted IS*2* translocatable unit (TU) is shown below.

Alternatively, the transposition mechanism of IS*2* elements could explain the presence of multiple copies of the ~210 kb region. IS*2* and other IS*3* family members utilize a “copy-out-paste-in” mechanism that involves formation of a circular double-stranded DNA structure [31,33,34]. The intermediate, often called a minicircle, is essentially a circularized version of a single IS*2* element that is capable of inserting itself into a new location. To determine whether high coverage of the ~210 kb region resulted from the production of a double-stranded circular molecule, we performed PCR analyses using primers that anneal near the ends of the ~210 kb region (Circ1 and Circ2, Fig 2A), such that they would amplify a ~2.5 kb product spanning the junction of the circularized element. Only one PCR product, 2.2 kb in size, was generated from WT *Bt*E264 cells (Fig 2B), and DNA sequence analysis of several independent PCR products indicated the junction contains IS*Bma*1b and one copy of *orfA* and *orfB* (more specifically, *orfB*_*β*_ based on the single nucleotide variant; Fig 2C). These data are consistent with the high coverage region corresponding to a ~210 kb “megacircle” that is formed by a reaction involving the sequence between the single nucleotide variation in *orfB* and the IRL of IS*2*β, most likely within the IRL of IS*2*α (since transposases typically bind to, and catalyze recombination within, inverted repeats; Fig 2D; [32,33,35,36]). The junction of the megacircle is different from those of *E*. *coli* IS*2* mini-circles where the inverted repeats are joined together and separated by a one- or two-base spacer [31,37], suggesting that the chemistry for the reaction involving the IS*2*-like elements in *Bt*E264 is different than what has been described for *E*. *coli* IS*2* elements. Multiple attempts to detect an IS*2* mini-circle junction in *Bt*E264 were unsuccessful, providing additional evidence for a distinct mechanism. Production of a circular DNA intermediate from two distant IS*2* elements has not been reported. These data suggest that the *Bt*E264 *bcpAIOB*-containing element may represent a previously undescribed IS*2*-containing composite transposon and mobile genetic element (MGE).

### The IS*2*-like megacircle of *Bt*E264 has features resembling the IS*26* translocatable unit

**Fig 3:**
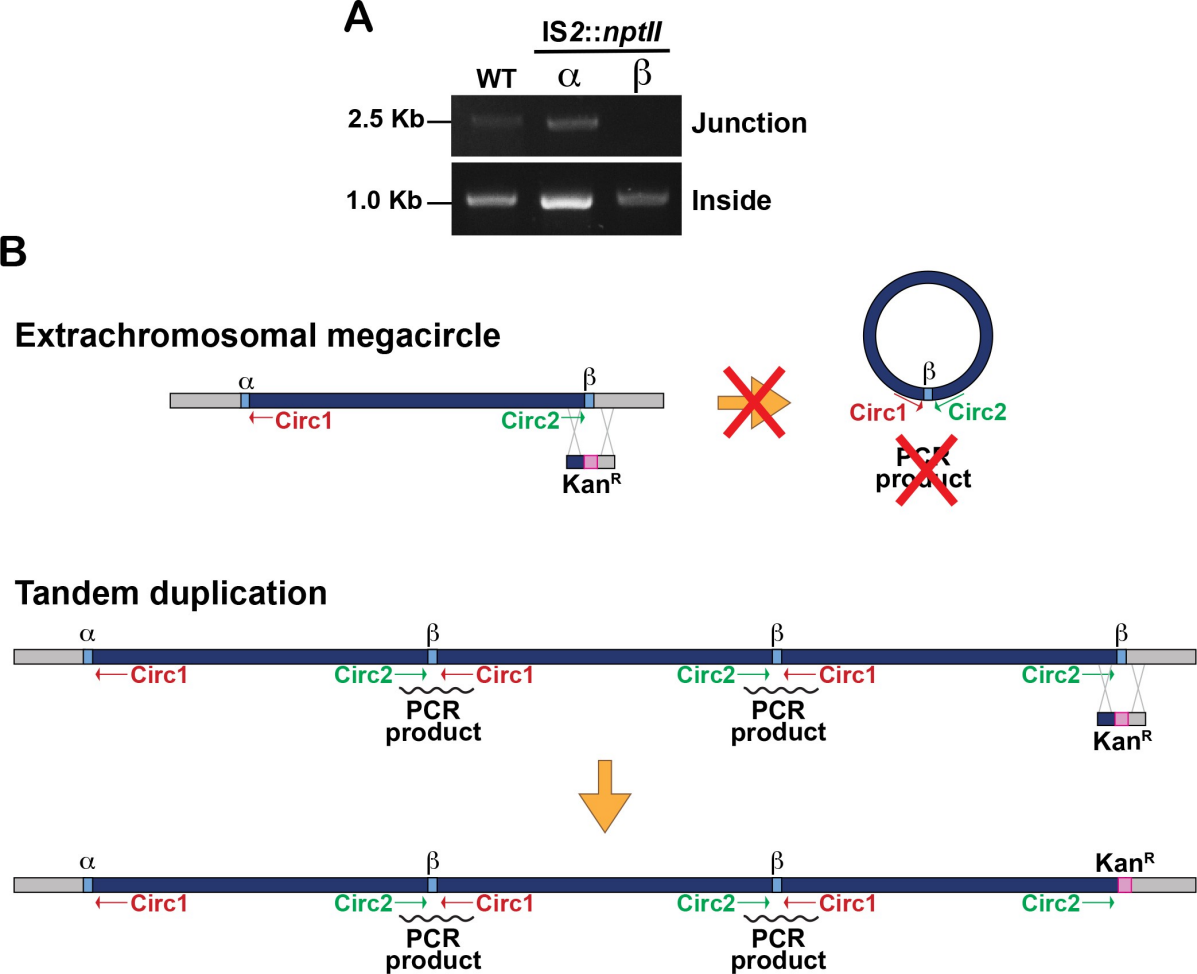
Megacircle production is disrupted in the absence of *orfAB*_*β*_. (**A**) PCR analyses to detect the megacircle junction using primers Circ1 and Circ2 (top), or primers that bind within the putative composite transposon (bottom), in WT and strains with defective IS*2*α or IS*2*β elements. During construction of IS*2*α::*nptII* and IS*2*β::*nptII*, several kanamycin-resistant colonies were analyzed; a representative strain is shown. (**B**) Graphical representation of detection (or lack of) of a PCR product with primers Circ1 and Circ2 from an extrachromosomal megacircle (top) compared to detection of a product from the interphase of multiple copies of the element present in tandem repeats.

Many transposases, including IS*3* family members, have a preference for acting on the element from which they were expressed (called *cis* activity), resulting from binding of the N-terminal domain of the nascent enzyme to its target sequence as it emerges from the ribosome [33,38]. The ends of the *bcpAIOB*-containing composite transposon are over 200 kb apart, yet they come together to form the megacircle. To determine the contribution of each IS*2*-like element to megacircle synthesis, we constructed mutant strains in which *orfAB* from IS*2*α or IS*2*β was replaced by double recombination with *nptII*, encoding kanamycin resistance. The inverted repeats, where transposase-mediated recombination is expected to occur, were left intact. The megacircle junction was detected by PCR in the IS*2*α::*nptII* strain, but not in the IS*2*β::*nptII* strain, indicating that the megacircle-forming reaction is a transposase-dependent event that requires the transposase encoded by IS*2*β, but not that encoded by IS*2*α (Fig 3A).

Lack of detection of a PCR product with primers Circ1 and Circ2 in the IS*2*β::*nptII* mutant provides additional evidence that the increased NGS coverage is due to the presence of extrachromosomal megacircle molecules and not tandem copies of the 210 kb region. If tandem repeats of the element were present in the genome (approximately 3 copies based on the NGS coverage), then they would each be separated by one copy of IS*2*β, based on our sequence analysis of the PCR product generated by Circ1 and Circ2 (Fig 3B). Mutation of the IS*2*β at the junction with the rest of the chromosome (the one farthest to the right in Fig 3B) would not abrogate amplification of PCR products with primers Circ1 and Circ2 as those junctions would still be present between the tandem copies (Fig 3B, bottom panel). Together, therefore, our results provide strong evidence that the *bcpAIOB* operon is located within a transposable element that forms an extrachromosomal circular intermediate.

Our data suggest that, despite lack of similarity between the transposases, the IS*2-*dependent megacircle bears mechanistic similarity with IS*26*-containing composite transposons. In its stationary form, a typical IS*26* composite transposon is composed of two IS*26* elements positioned in direct orientation and flanking passenger DNA that contains genes encoding antibiotic resistance [39–41]. IS*26* does not appear to transpose as a single IS element [42], instead, it mobilizes via a circular molecule called a translocatable unit (TU). Formation of the TU is mediated by the transposase encoded by the “left” IS*26* (with the IS elements oriented such that the transposases are encoded left to right), and the TU is composed of the “left” IS*26* element and passenger DNA [42–44]. Thus, both the *Bt*E264 IS*2-*like transposase and the IS*26* transposase appear to catalyze reactions that involve distantly-located ISs to create circular intermediates that contain only the “left” IS element and the DNA intervening between the ISs (Fig 2D).

### Formation of the IS*2*-like megacircle correlates with BcpA activity and CDS

**Fig 4:**
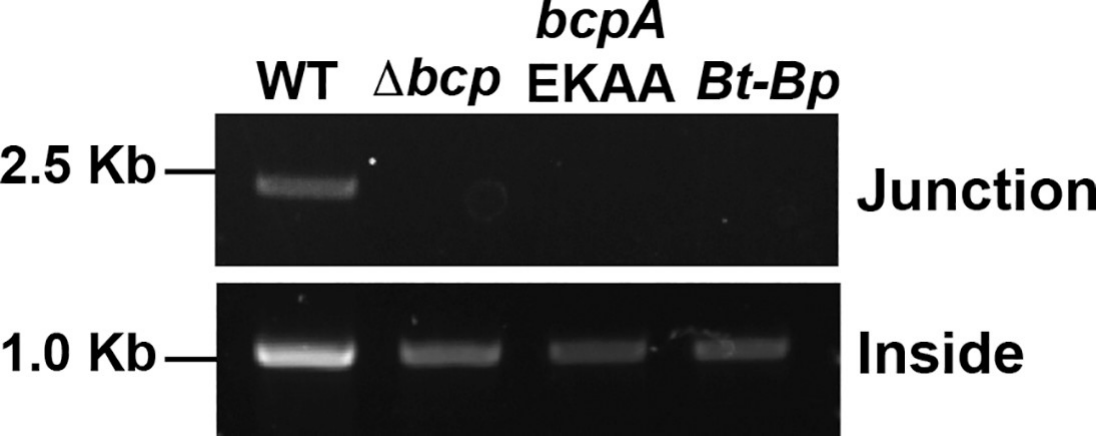
PCR analyses to detect the megacircle junction in *Bt*E264 mutant strains. Detection of the megacircle junction using primers Circ1 and Circ2 (top) or primers that bind within the putative composite transposon (bottom) in WT and mutant strains lacking the *bcpAIOB* locus (Δ*bcp*) or producing catalytically inactive (BcpA_EKAA_) or chimeric (*Bt*-*bp*) BcpA.

In addition to WT *Bt*E264, our NGS studies included the Δ*bcpAIOB* mutant, and for this strain increased coverage in chromosome I was not observed. PCR with Circ1 and Circ2 failed to produce a product from this strain, as well as from the BcpA_EKAA_ strain, which produces a catalytically inactive BcpA protein (Fig 4). The Δ*bcpAIOB* and BcpA_EKAA_ mutants are defective for both CDI and CDS. We showed previously that a chimeric BcpA protein, in which the conserved region from the *Bt*E264 allele is fused to the variable catalytic BcpA-CT region from an allele present in *B*. *pseudomallei* 1106a, can mediate inhibition of neighboring susceptible cells via CDI, but cannot mediate CDS [24,26]. The megacircle junction was not detected by PCR with Circ1 and Circ2 in the strain producing the chimeric BcpA protein (*Bt-Bp*, Fig 4). These data indicate a positive correlation between megacircle formation and CDS phenotypes.

### Megacircle formation is required for community-associated (CDS) phenotypes

**Fig 5:**
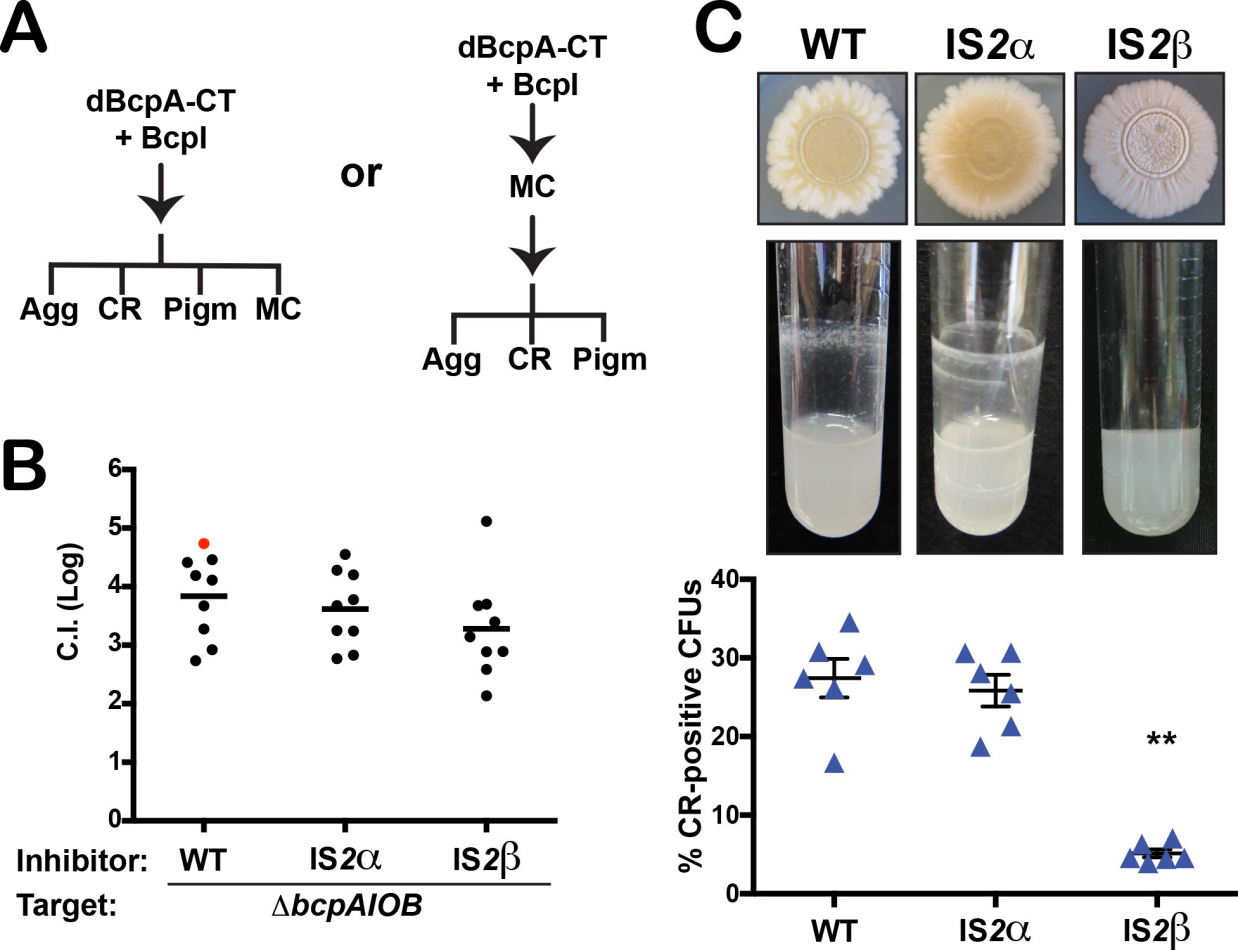
Megacircle production and community-associated behaviors, but not CDI, are disrupted in the absence of *orfAB*_β_. (**A**) Models illustrating the possible correlations between CDS and megacircle formation. Delivered C-term of the BcpA toxin, dBcpA-CT. (**B**) Contact-dependent growth inhibition (CDI) is not affected by disruption of IS*2*α or IS*2*β. Inhibitors (WT, IS*2*α::*nptII* or IS*2*β::*nptII*) were co-cultured with Δ*bcpAIOB* (a strain susceptible to killing via CDI) for 24 hours then plated on selective media to calculate the competitive index (C.I.). When only WT bacteria were recovered, the data point is displayed in red; in this case the actual C.I. is greater than or equal to the represented value. Differences in C.I. are not statistically significant. (**C**) Ability of WT, IS*2*α::*nptII* or IS*2*β::*nptII* to display phenotypes linked to community-associated behaviors. These phenotypes include: production of pigments by colony biofilms (top), autoaggregation in M63 minimal medium (middle), or the proportion of Congo red (CR) binding colonies. *P* values were obtained using Mann-Whitney U test comparing mutant strains to WT. Results are shown as mean +/- SEM of three independent experiments (*n* = 6). **P < 0.01.

A possible explanation for the link between the megacircle and CDS phenotypes is that circularization of the element is a result of interbacterial signaling that leads to changes in gene expression in a cell that has received a BcpA-CT from a neighboring cell, i.e., megacircle formation is a newly-identified CDS phenotype (Fig 5A, left hypothesis). Alternatively, the CDS phenotypes could be a consequence of megacircle production via an unknown mechanism (Fig 5A, right hypothesis). Characterization of the strain in which IS*2*β was replaced with *nptII* showed that these bacteria have a functional BcpAIOB system that can mediate CDI (it outcompeted the Δ*bcpAIOB* strain as well as wild-type bacteria, Fig 5B), but that cannot mediate CDS (it did not aggregate in minimal media, bind Congo red or produce the yellow/brown pigment, Fig 5C). Our data indicate, therefore, that BcpA activity and IS*2*β are both necessary for formation of the megacircle, and that the extrachromosomal megacircle molecule is somehow required for CDS phenotypes (Fig 5A, right hypothesis).

### The *bcpAIOB*-containing element is capable of mobilization to a new chromosomal location

**Fig 6:**
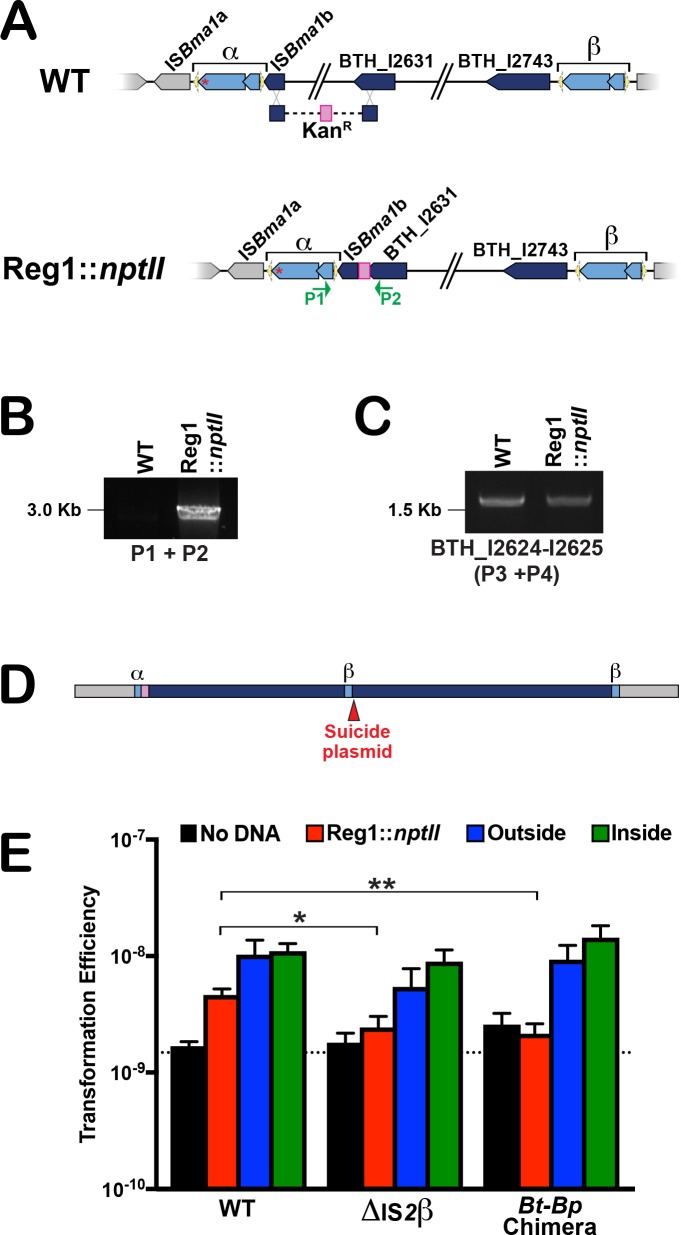
Evidence for intracellular movement of the IS*2*-like megacircle. (**A**) Diagram depicting the strategy used to replace the first ~48 kb (Region 1) of the *bcpAIOB*-containing putative composite transposon (dark blue) with a cassette containing the gene that confers kanamycin resistance, *nptII*, and FRT-binding sites (pink). (**B**) PCR analyses to confirm removal of Region 1 in strain Reg1::*nptII* using primers P1 and P2 (shown as green arrows in panel A) which are close enough to generate a product only after deletion of the ~48 kb region. (**C**) PCR analyses of WT *Bt*E264 and strain Reg1::*nptII* using primers P7 and P8, which would amplify two genes from within the Region 1 sequence. (**D**) Integration of a suicide plasmid (red arrowhead) within the Region 1 sequence of the mobilized element, followed by plasmid rescue studies, suggests that the mobilized megacircle inserted adjacent to the truncated composite transposon carrying Reg1::*nptII* mutation. (**E**) Comparison of the transformation efficiencies upon deletion of Region 1 in strains that are positive (WT) or deficient (ΔIS*2*β and *Bt*-*Bp* chimera) in the production of the megacircle. No DNA, black bars; DNA to introduce the Reg1::*nptII* mutation, red bars; DNA to introduce an *nptII* cassette outside of the putative composite transposon, blue bars; DNA to introduce an *nptII* cassette inside the putative composite transposon, green bars. Horizontal dashed line represents the average lowest limit of detection. *P* values were obtained using Mann-Whitney U test comparing mutant strains to WT when the same DNA (or no DNA) was added. Results are shown as mean +/- SEM of three independent experiments with three technical replicates each (*n* = 9). *P < 0.05; **P < 0.01.

Our data indicate that the *bcpAIOB*-containing element forms extrachromosomal circular DNA molecules in an IS*2*β-dependent manner and that these are conceptually similar to the TUs formed by IS*26*. Our initial approach to determine if the megacircle functions as a TU was to determine if it could be transferred between cells, and we reasoned that the optimum recipient strain for testing this hypothesis would be one lacking the entire ~210 kb element. Although the element contains several genes that encode proteins that are expected to be necessary for viability (S1 Table), the essentiality of those genes has not been tested in *Bt*E264. We therefore set out to delete the element (simultaneously testing the essentiality of genes within it), and we began by replacing the ~48 kb region encompassing genes BTH_I2587-I2630 (which we refer to as Region 1, or Reg 1), with an *nptII*-containing cassette, taking advantage of the fact that *B*. *thailandensis* is naturally competent and proficient at homologous recombination (Fig 6A). PCR analyses of the resulting kanamycin-resistant transformants with primers P1 and P2, which bind to BTH_I2585 (*orfA*_α_) and BTH_I2631, respectively (Fig 6A), yielded the expected ~3 kb DNA fragment (Fig 6B). However, PCR analyses to amplify genes BTH_I2604-2605, located within Reg1, yielded a DNA fragment that was identical in size to that amplified in WT *Bt*264 (Fig 6C), indicating the presence of those genes. The simplest explanation for this result is that the *bcpAIOB*-containing megacircle is, in fact, a translocatable unit that integrated into a new site in the chromosome (i.e., it had mobilized intracellularly) during the construction of the Reg1::*nptII* mutant because one or more genes within Reg1 are, in fact, essential.

To test the hypothesis that the megacircle had integrated at a new site, we used plasmid rescue to determine its new location by identifying the DNA sequence adjacent to BTH_I2587 (Fig 1B). Briefly, we constructed a suicide plasmid containing a 500 bp DNA fragment corresponding to the 5′ end of BTH_I2587 (a sequence unique to the mobilized IS*2* element), introduced the plasmid into the Reg1::*nptII* mutant and selected TMP-resistant cointegrants. Genomic DNA isolated from two independent cointegrants was subjected to restriction digestion, then the gDNA fragments were ligated and transformed into *E*. *coli*, followed by selection of TMP-resistant transformants. The plasmids recovered contained a ~28 kb sequence identical to the “*β* end” of the *bcpAIOB*-containing element (Figs 1B and 6D), indicating that the megacircle had integrated in tandem to the Reg1::*nptII*-containing element during deletion of Reg1. This result demonstrates the ability of the megacircle to integrate into the chromosome, indicating that the megacircle is a TU and that the *bcpAIOB*-containing element is a newly identified composite transposon and mobile genetic element.

The Reg1::*nptII* merodiploid strain provided an opportunity to obtain additional evidence that the 210 kb *bcpAIOB*-containing region forms an extrachromosomal circular DNA molecule. We removed the *nptII* gene from the Reg1::*nptII* strain by Flp recombinase mediated recombination to create Reg1-Kan^S^, which was then incubated with a linear DNA molecule containing the *nptII* gene flanked by 500 bp sequences corresponding to those 5′ and 3′ to *bcpAIOB*. PCR analyses indicated that one copy of *bcpAIOB* was replaced with *nptII* in the resulting Km^R^ transformants, while one copy of *bcpAIOB* remained intact (S4 Fig). Only when the second copy of *bcpAIOB* was replaced with *nptII* (after removal of *nptII* by Flp recombinase from the site of the first replacement), were the *bcpAIOB* genes undetectable by PCR (S4 Fig). By contrast, when wild-type *Bt*E264 was incubated with the same linear DNA molecule, only primers corresponding to replacement of *bcpAIOB* with *nptII* yielded a PCR product–the *bcpAIOB* genes were undetectable in this strain (S4 Fig). Thus, despite wild-type *Bt*E264 containing, on average, about three copies of the 210 kb region, only one copy (the chromosomal copy) is stably maintained in the cell and susceptible to mutagenesis that is heritably maintained. These data provide further support for the 210 kb *bcpAIOB*-containing region existing as an extrachromosomal circular molecule. Moreover, they support the prediction, based on the absence of an identifiable *ori*, that the megacircle is incapable of replication, explaining why it is possible to construct strains with mutations within the element by allelic exchange, despite the presence of multiple copies of target genes within the cell at any given time; although recombination with the megacircle may occur, such recombinants cannot be selected as the megacircle is essentially a suicide plasmid.

The efficiency of obtaining kanamycin-resistant transformants upon exposure of *Bt*E264 cells to DNA that replaces Reg1 with *nptII* can be used to measure megacircle-dependent mobilization of the element. In WT *Bt*E264, transformation efficiency for the introduction of the Reg1::*nptII* mutation was 4.61 x10^-9^ (Fig 6E). By contrast, for the mutant lacking *orfAB*_*β*_ and the strain producing the *Bt-Bp* BcpA chimera (which do not produce megacircles, Figs 3A and 4), the number of kanamycin-resistant colonies obtained was either zero or not significantly different from the number obtained when no DNA was included (note that the average limit of detection for this assay is 1.49 x 10^−9^). To rule out the possibility that the low transformation efficiency observed with the megacircle-deficient strains was caused by a defect in competence, we introduced a cassette carrying *nptII* by natural transformation using DNA fragments with homology corresponding to regions inside or outside the ~210 kb MGE. A similar number of transformants was obtained for these cassettes in all strains (Fig 6E), indicating that competence, or downstream processes that allow natural transformation of cells, are not affected in megacircle-deficient strains. Together, these data strongly support the conclusion that translocation of the *bcpAIOB*-containing MGE occurs via the megacircle.

**Fig 7:**
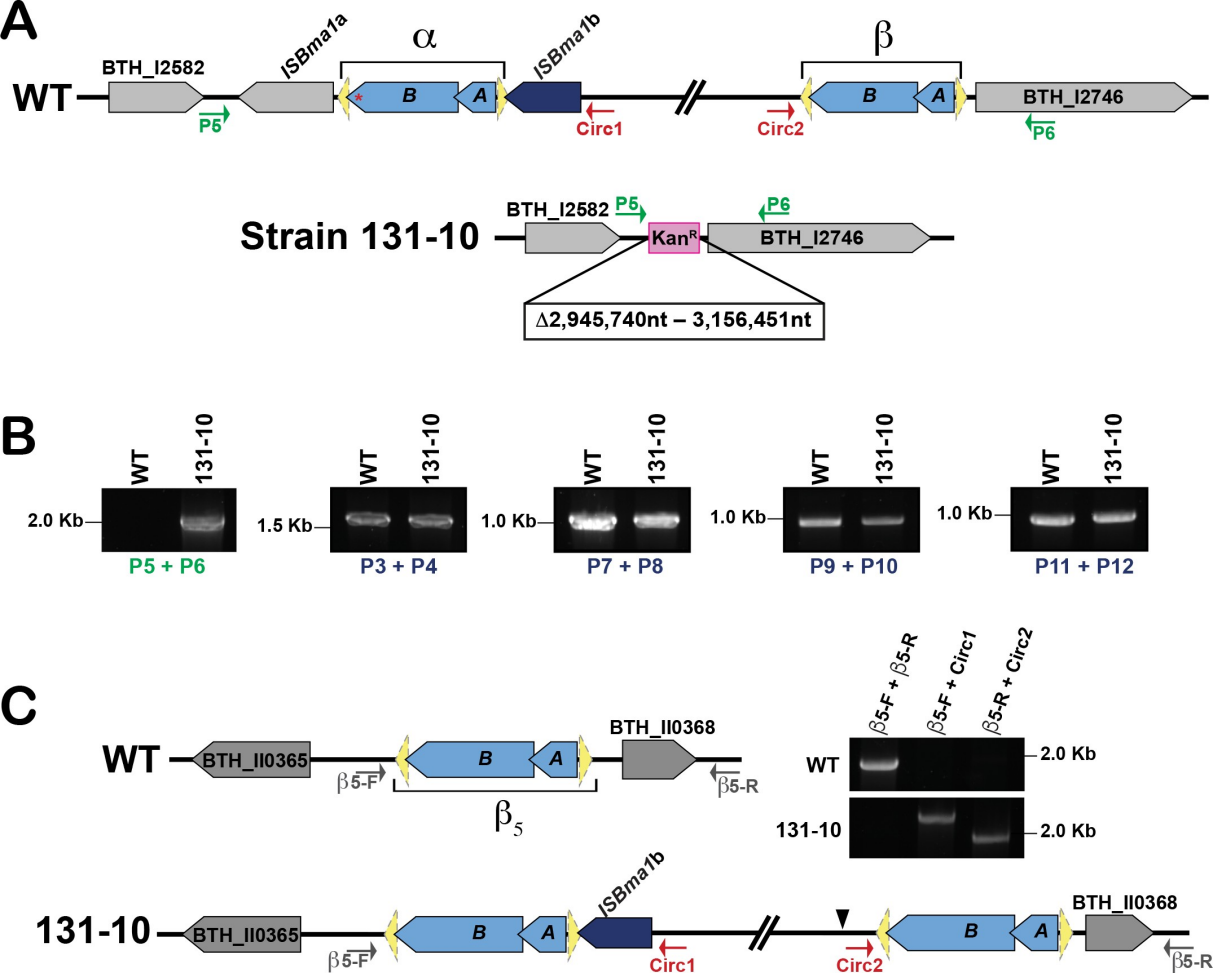
The IS*2*-like Translocatable unit mobilized to chromosome II in the 131–10 strain. (**A**) Graphical representation of the region in chromosome 1 where the *bcpAIOB*-containing composite transposon is located (top). Graphical representation of strain 131–10 in which the entire composite transposon has been replaced by an *nptII* cassette (bottom). (**B**) PCR analyses to confirm removal of the MGE in strain 131–10 using primers P5 and P6 (shown as green arrows in panel A). PCR analyses detect DNA corresponding to genes within the composite transposon in strain 131–10. (**C**) IS*2*β_5_ in chromosome II in a WT background (top), or upon insertion of the IS*2*-like translocatable unit (bottom). PCR analyses of WT and strain 131–10 to confirm the insertion of the IS*2*-like translocatable unit into chromosome II. The distance between the binding site of primers *β*5-F and *β*5-R (grey arrows) is approximately 1.8 kb in WT DNA. Primers Circ1 and Circ2 (red arrows) bind specifically to the ends of the mobilized *bcpAIOB*-containing element. The insertion site of pABT73-TMP is denoted with a black arrowhead.

We continued to delete the rest of the sequences within the *bcpAIOB*-containing MGE despite the presence of a tandem copy. The final step used regions of homology corresponding to genes outside the element, BTH_I2582 and BTH_I2746 (Fig 1B). PCR analyses of the resulting kanamycin-resistant strain (131–10) with primers P5 and P6 confirmed that nucleotides 2,945,740 to 3,156,451 had been replaced with the *nptII* cassette (Fig 7A and 7B). However, as we now expected, genes within the MGE were still present, as evident from PCR amplification of DNA fragments from different regions of the element (Fig 7B and S4 Fig). To determine the location of the element in this strain, we again used a plasmid rescue approach, this time with the suicide plasmid containing DNA corresponding to the 3′ end of BTH_I2743. Sequence analysis of the recovered plasmids indicated that the adjacent DNA corresponded to the 5′ end of BTH_II0368 on chromosome II, the gene adjacent to IS2β_5_ (BTH_II0366-0367; Fig 1A and 7C and S2 Fig). PCR analysis of strain 131–10 with primers that anneal to regions adjacent to BTH_II0365 and BTH_II0368, paired with primers Circ1 and Circ2, respectively, confirmed the MGE translocation (Fig 7C). Furthermore, the element was functional from its new location as the megacircle was detectable in strain 131–10 by PCR, and CDS phenotypes were produced (S5 Fig). These data, showing that the megacircle can integrate into a different chromosome, provide additional proof that the *bcpAIOB*-containing element is a composite transposon that is mobile via an extrachromosomal translocatable unit. PCR analyses of WT *Bt*E264 with primers flanking IS*2*β_2_, IS*2*β_3_, and IS*2*β_4_ indicate that these regions are as indicated by the reference genome sequence, corroborating our findings that multiple copies of the ~210 kb region are due to extrachromosomal DNA molecules and not to an MGE integrated next to an IS*2*-like element (S6 Fig).

The 5-bp target duplication characteristic of mobilized IS*2* elements was not observed in the plasmids recovered from strains Reg1::*nptII* and 131–10. Integration of the megacircle next to an existing IS*2* (IS*2*β in strain Reg1::*nptII* and IS2β_5_ in strain 131–10) could result from homologous recombination, and we cannot rule out this possibility because introduction of the Reg1::*nptII* mutation, a process that requires a functional recombination machinery, has been our method of selection for bacteria with a mobilized megacircle. However, taking into account the mechanistic similarities between the IS*26* TU and the megacircle, it is plausible that integration was transposase mediated. Frequency of transposase-dependent IS*26* TU integration via the targeted conservative mechanism (which requires a pre-existing IS*26* element at the target site and does not generate a target duplication) is much higher than random transposase-dependent integration (which results in duplication of the element and generation of the target duplication) or Rec-dependent cointegrant formation [42,44,45].

## Discussion

In this study, we identified a ~210 kb mobile genetic element within chromosome I of *B*. *thailandensis* E264 (*Bt*E264) that contains the *bcpAIOB* genes, has IS*2*-like ISs at each end and defines a previously unknown class of IS*2*-containing composite transposon. Our data indicate that the transposon moves by a copy-out-paste-in mechanism that utilizes a double-stranded circular DNA intermediate, which we refer to as the megacircle. Only the “left” IS*2*-like element (IS*2*β) is required for megacircle formation, and only IS*2*β is present in the megacircle. We also showed that mobilization of the transposon to a new location within the *Bt*E264 genome occurred next to a pre-existing IS*2*-like element, thus recreating the composite transposon architecture. Our data also show that megacircle formation is required for CDS phenotypes, and that in addition to IS*2*β, creation of the megacircle requires BcpA activity.

IS elements, the simplest transposable units in bacterial genomes, are composed of one or two transposase-encoding genes flanked by inverted repeats that serve as transposase binding sites [33]. Mobility of IS*2* in *E*. *coli* occurs through a copy-out-paste-in mechanism consisting of production and integration of a double-stranded DNA (dsDNA) minicircle intermediate [31,37,46–48]. During IS*2* minicircle biogenesis (the copy-out step), an active OrfAB transposase binds and cleaves the IS to generate the characteristic figure-eight structure that becomes the IS*2* minicircle upon DNA replication and repair. The second step of transposition, integration of the minicircle at a target site (the paste-in step), begins with increased *orfAB* expression from a strong promoter formed by the abutted end repeats and spacer located in the minicircle junction. It ends with *cis* activity of the transposase that results in cleavage of the abutted ends to generate a reactive linear IS that can integrate into a dsDNA target site [31,49]. No definitive insertion sequence specificity has been identified for *E*. *coli* IS*2*, however, integration of the minicircle is not random, as it occurs in regions where the host DNA structure shifts due to abrupt changes in GC skew [50].

The predicted amino acid sequence of OrfAB encoded by the IS elements flanking the *bcpAIOB*-containing MGE in *Bt*E264 is highly similar to that of IS*2* OrfAB from *E*. *coli*, especially within the predicted DNA binding and catalytic domains (S1 Fig) [47]. However, we have obtained no evidence that any of the IS*2*-like elements in *Bt*E264 function independently as an IS. Instead, our data indicate that IS*2*α and IS*2*β function together as a composite transposon that mobilizes via an extrachromosomal megacircle containing 158 ORFs, including the *bcpAIOB* genes. Although IS*2* from *E*. *coli* and the IS*2*-like elements in *Bt*E264 both form circular intermediates, the contents of the intermediates are different. Understanding why the transposase acts within one element in the *E*. *coli* IS and between separate elements in the *Bt*E264 transposon awaits further investigation. However, as IS*2*-containing composite transposons have not been reported previously, the element in *Bt*E264 represents the first-identified member of this class of transposon.

IS*26* elements, which are members of the IS*6* family, play critical roles in the dissemination of genes encoding antibiotic resistance in Gram-negative bacteria [51–54]. IS*26*-containing transposons have been shown to move via an excised circular element called a translocatable unit (TU, [43]). The composition of the TU is analogous to that of the *Bt*E264 megacircle, it contains one IS plus the DNA intervening between the two IS elements in the composite transposon. Also, similar to the case for the *Bt*E264 IS*2*-like element-containing transposon, only the “left” IS*26* is required for TU formation and it is the “left” IS*26* that is included in the TU [43]. Moreover, it also appears that IS*26* elements do not transpose as single IS elements [42]. Thus, although the *Bt*E264 IS*2*-like and IS*26* transposases share only limited amino acid similarity (S1 Fig), they both appear to catalyze reactions that involve distantly-located sequences to create complex, extrachromosomal circular transposition intermediates.

Integration of an IS*26*-containing TU into a target molecule can occur randomly via an untargeted replicative mechanism involving duplication of IS*26* and the target sequence, or via a targeted conservative mechanism that targets an existing IS*26* element to recreate an IS*26*-containing composite transposon without duplication of any sequence [41,42,44,45]. The targeted conservative mechanism can occur by homologous recombination between sequences within the IS*26* elements or, much more efficiently, by transposase-catalyzed recombination between sequences at the left or right ends of the IS*26* elements [42]. For targeted integration involving transposase-catalyzed recombination, both IS*26*-encoded transposases must be active [43]. The fact that a composite transposon that is apparently identical to the one present in WT *Bt*E264 was recreated in chromosome II in strain 131–10, or adjacent to the partially deleted MGE in the Reg1::*nptII* mutant, provides evidence that the *Bt*E264 megacircle is capable of transposition via a targeted conservative mechanism, similar to the IS*26*-containing TU. Whether integration of the megacircle occurred via RecA-dependent or transposase-mediated recombination is currently unknown and under investigation. If it occurred via transposase-mediated recombination it would suggest that the recently discovered targeted reaction mediated by the IS*26*-encoded transposase is used by multiple transposases, including those with surprising little amino acid similarity.

Targeted conservative transposition of IS*26*-containing transposons carrying genes encoding *β*-lactamases facilitates amplification of the element in response to exposure of the bacterium to *β*-lactams [55]. Such a response to selective pressure, resulting in multiple copies of the transposon in tandem array, could explain the integration of the megacircle in strain Reg1::*nptII* (since Region 1 apparently contains genes essential for cell growth). In addition to tRNA-synthetases and other predicted housekeeping proteins, BcpI (the immunity protein) is essential in bacteria producing a functional BcpA protein. Interestingly, a *Bt*E264 transposon mutant library constructed by Gallagher *et al*. includes mutants with transposons inserted within genes that span the *bcpAIOB*-containing MGE that are predicted to be essential, including *bcpI* [56]. Our data suggest that construction of these mutants may have been possible due to the presence and mobilization of the *bcpAIOB*-containing MGE.

We showed previously that a catalytically active BcpA protein is required for changes in gene expression that lead to behaviors such as biofilm formation and pigment production, a phenomenon we call CDS [26]. The mechanistic link between BcpA activity and gene expression changes, however, is unknown. We found in this study that production of the megacircle correlated directly with BcpA activity, suggesting that megacircle formation is another CDS phenotype. However, deletion of *orfAB* in IS*2*β resulted in not only lack of megacircle formation, but also lack of other CDS phenotypes, despite the BcpAIOB proteins being unaltered and functional (as evident by the fact that the ΔIS*2*β strain was capable of mediating CDI). These data suggest a linear relationship between BcpA activity, megacircle formation, and CDS phenotypes (i.e., active BcpA induces megacircle formation and megacircles induce CDS phenotypes). While understanding the mechanism by which megacircles induce CDS phenotypes will require further investigation, one possibility is that megacircle-dependent gene expression changes result simply from increased gene copy number. Consistent with this hypothesis, transcriptomic analyses of *Bt*E264 cultured under CDS-inducing conditions revealed increased expression of 58 of the 161 genes within the composite transposon [26]. While some of these genes contribute directly to CDS phenotypes, such as the *csu* operon which is involved in biofilm formation, others, such as those predicted to encode regulatory factors, may function indirectly.

The mechanism by which BcpA induces megacircle formation is similarly unknown. Absence of megacircles in the strain producing chimeric BcpA (*Bt-Bp*, Fig 4) indicates that the correct catalytic activity (i.e., that of the BcpA protein encoded by the *Bt*E264 allele) is required. One hypothesis is that the C-terminus of BcpA in *Bt*E264, which is predicted to share structural similarity with holiday junction resolvases, is directly involved in the recombination reaction mediated by the IS*2*β-encoded transposase. Another possibility is that activity of the BcpA C-terminus results in a shift from production of OrfA, which inhibits activity of the IS*2* element-encoded transposase in *E*. *coli*, to production of the full-length OrfAB transposase [31,32]. We are currently investigating these possibilities, and can also envisage others.

Regardless of the underlying mechanisms, megacircle formation is clearly a result of contact-dependent interactions between cells producing the same BcpAIOB proteins. We hypothesize that CDI/CDS systems function as both harming and helping greenbeards, inhibiting the growth of non-self bacteria, and inducing cooperative behaviors in self bacteria, upon cell-cell contact, with self defined by the specific *bcpAIOB* (or *cdiBAI*) allele [14]. Our current results provide evidence that the *bcpAIOB* genes in *Bt*E264 are located within a mobile genetic element, which would put the *bcpAIOB* genes and others encoding proteins involved in cooperative behaviors in linkage disequilibrium with the rest of the chromosome, another feature of greenbeard genes. But can the *bcpAIOB*-containing MGE translocate from one cell to another? Our data suggest that the recipient cell would have to contain at least one IS*2*-like element for the megacircle to recreate the transposon. Bioinformatic analyses indicate the presence of highly conserved IS*2*-like elements in other *Burkholderia* species, including members of the *Burkholderia cepacia* complex (Bcc). In addition, the recipient cell would have to produce the correct outer membrane receptor and cytoplasmic membrane translocation protein for CDS to occur. Although the identities of these proteins for BcpA_E264_ are unknown, we showed recently that *B*. *dolosa* strain *Bd*AU0158 can mediate CDI using BcpAIOB proteins that are nearly identical to those produced by *Bt*E264 [57], and that *Bd*AU0158 can induce CDS phenotypes in a *Bt*E264 Δ*bcpA* mutant [26], suggesting that these strains share the receptor and translocator proteins. The recipient cell would also have to tolerate duplicate copies of the essential genes on the MGE, or have a mechanism for deleting or mutating them.

Genomic analyses have predicted that CDI/CDS system-encoding genes are located within genomic islands [58,59]. In search of evidence for transfer of *bcpAIOB* genes among *Burkholderia* species via horizontal gene transfer, we recently searched for *bcpAIOB* homologs and then used Mauve software to detect evidence of synteny surrounding those genes [57]. Our search identified 13 strains and six *bcpAIOB* alleles that differed only in the regions encoding the C-terminal ~100 aa of BcpA and the N-terminal ~150 aa of BcpI. Flanking genes were similar only in strains that contained the same allele–there was no synteny around the *bcpAIOB* genes among strains with slightly different *bcpAIOB* alleles [57]. These data suggest that if these closely-related alleles were acquired horizontally, there has been substantial evolution since that time (i.e., they do not appear to be located within the same or similar genomic islands currently). However, further comparison of the three genomes containing *bcpAIOB* alleles identical to that in *Bt*E264 revealed that although the entire genomes appear to be nearly identical, strain *Bt*E254 lacks all three IS elements (IS*Bma*1a, IS*2*α, and IS*Bma*1b) at the “*α* end” of the *bcpAIOB*-containing transposon. Moreover, these elements are flanked by direct repeats in *Bt*E264, and there is no apparent “scar” in *Bt*E254 (S7 Fig), suggesting that *Bt*E264 gained these IS elements, rather than *Bt*E254 losing them. It appears, therefore, that a relatively recent transposition event introducing IS*Bma*1a, IS*2*α, and IS*Bma*1b into chromosome I of *Bt*E264 resulted in the formation of the *bcpAIOB*-containing IS*2*-like composite transposon, which is currently mobile, at least intracellularly.

If it occurs, interbacterial transfer of the MGE would support the selfish gene hypothesis for *bcpAIOB*. Our data indicate that *B*. *thailandensis* communities are composed of megacircle-producing bacteria. A non-self bacterium (one that does not contain the same *bcpAIOB* allele) that encounters such a community may receive a BcpA C-terminus and be killed due to the lack of the correct BcpI protein. Alternatively, the invader may receive the megacircle, and if the megacircle can insert into the chromosome, the invader can be converted into a ‘self’ cell that is not only immune to BcpA-mediated CDI, but that could produce megacircles and, consequently, proteins involved in cooperative behaviors. If the invading bacterium contains a different *bcpAIOB* allele, or another mechanism to kill the initial community, the invading bacterium and its descendants will eliminate the resident population and take over the niche, and the selfish *bcpAIOB* genes will propagate within the newly established population. Experiments to determine if the *bcpAIOB*-containing MGE can be transferred intercellularly are underway.

## Methods

### Bacterial strains and plasmids

*Burkholderia thailandensis* E264 is an environmental isolate [60]. All plasmids and strains used in this study are listed in S2 Table in Supporting Information. Plasmids were maintained in *E*. *coli* DH5*α* and introduced into *Bt*E264 through biparental matings using *E*. *coli* RHO3 as the plasmid donor [61,62]. *Bt*E264 and *E*. *coli* strains were grown overnight with aeration at 37°C (unless indicated) in low salt Luria-Bertani (LSLB, 0.5% NaCl). Antibiotics were added to cultures at the following concentrations: 250 μg/mL (for *Bt*E264) or 50 μg/mL (for *E*. *coli*) kanamycin (Kan), 100 μg/mL ampicillin, 200 μg/mL (for *Bt*E264) or 50 μg/mL (for *E*. *coli*) trimethoprim (TMP), or 200 μg/mL diaminopimelic acid as appropriate. When indicated, *Bt*E264 was cultured on M63 minimal medium (110 mM KH_2_PO_4_, 200 mM K_2_HPO_4_, 75 mM (NH_4_)_2_SO_4_, 16 nM FeSO_4_) supplemented with 1mM MgSO_4_ and 0.2% glucose [63].

### Construction of plasmids and mutant strains

*Bt*E264 IS*2*α::*nptII* and IS*2*β::*nptII* were constructed by natural transformation [63]. First, a 1.4 kb DNA fragment consisting of the gene encoding kanamycin resistance, its promoter, and flanking FRT sites was amplified from pUC18miniTn7(Km) by PCR using primers containing 5’ NdeI or EcoRV restriction sites. The DNA fragment was then introduced into the blunt cloning site of pJET1.2 (Thermo Fisher), resulting in plasmids pABT62-NdeI or pABT62-EcoRV. Additionally, DNA fragments around 750 bps (for IS*2*β) or 1.5 kb (for IS*2*α) in size, and 5’ or 3’ to the IS*2* elements, were amplified using *Bt*E264 genomic DNA as template. SOEing mutagenesis was then employed to construct a single DNA product in which the 5’ and 3’ regions of homology were joined and a NdeI site was added to the middle of the PCR product. Next, the fused DNA PCR product was cloned into the blunt cloning site of pJET1.2. The resulting plasmid was confirmed by Sanger sequencing and then subjected to linearization with NdeI (NEB), so that FRT-*nptII*-FRT (dropped from pABT62-NdeI with the same enzyme) could be cloned into the appropriate restriction site. This gave rise to plasmids pABT78 (IS*2*α::*nptII*) and pABT66 (IS*2*β::*nptII*); which in turn were linearized with HindIII (NEB) and transformed into *Bt*E264 WT.

Deletion of nucleotides 2,945,740 to 3,156,451 was achieved through a multi-step process involving natural transformation. First, ~750 nucleotides corresponding to the 5’ end of IS*Bma*1b or the 3’ end of BTH_I2631 were amplified from *Bt*E264 gDNA. Overlap PCR was performed to join the 5’ and 3’ homology sequences and form a single DNA product which included an EcoRV site in the middle. FRT-*nptII*-FRT (dropped from pABT62-EcoRV with the same enzyme) was then inserted into the EcoRV site of the fused sequences, generating plasmid pABT63 (Reg1::*nptII*). pABT63 was then introduced into WT *Bt*E264 cells by natural transformation followed by selection with kanamycin. The kanamycin cassette was removed from *Bt*Reg1::*nptII* transformants by Flp-*FRT* recombination using pFlpTet [25,64]. The same method was used to create pABT65 (Reg3::*nptII*) which included the sequence 5’ to BTH_I2671 or 3’ to BTH_I2705. pABT65 was then introduced into the kanamycin sensitive *Bt*Reg1 strain; the resulting *Bt*Reg1Region3::*nptII* transformants was then subjected to Flp-*FRT* recombination to generate a kanamycin sensitive version. Next, pABT68 (Reg4::*nptII*, generated by joining the sequence 5’ to gene BTH_I2706 and the sequence 3’ to IS*2*β) was used to construct *Bt*Reg1Region3-IS*2*β::*nptII* via natural transformation using the *Bt*Reg1Region3 kanamycin sensitive strain. The kanamycin cassette was then removed, resulting in *Bt*Reg1Region3-IS*2*β Kan^S^. Lastly, the sequence 5’ to IS*2*β and 3’ to IS*2*β was used to create pABT71 (Reg1-4::*nptII*), which was introduced into the *Bt*Reg1 Region3-ΔIS*2*β Kan^S^ strain to construct the final *Bt*E264 mutant lacking the *bcpAIOB*-containing mobile genetic element at its native location (strain 131–10). The kanamycin sensitive *Bt*Reg1 strain was also subjected to natural transformation with linear *bcpAIOB*::*nptII* gDNA obtained from strain Δ*bcpAIOB* (8) resulting in strain Reg1 *bcpAIOB*^**+/–**^. The latter strain was then subjected to Flp-*FRT* recombination to generate a kanamycin sensitive version and the resulting strain was used for a second round of transformation to replace the second *bcpAIOB* copy with *nptII* (Reg1 *bcpAIOB*^**–/–**^).

The transformation efficiencies when a FRT-*nptII*-FRT cassette is introduced inside or outside of the composite transposon were determined using pABT77 and pABT79 respectively; these plasmids were constructed as follows. First, a ~1.0 kb DNA segment, with a naturally present EcoRV restriction site located ~4.5 kb upstream (outside) or ~30 kb downstream (inside) of IS*2*α, was amplified from *Bt*E264 gDNA and cloned into pJET1.2. Next, the FRT-*nptII*-FRT cassette, dropped from pABT62-EcoRV, was inserted into the linearized pJET containing the “inside” or “outside” segment, generating plasmids pABT77 (outside of MGE::*nptII*) and pABT79 (inside of MGE::*nptII*).

Lastly, the suicide plasmids pABT73-TMP and pABT74-TMP were constructed as follows. Approximately 500 nucleotides were amplified from WT *Bt*E264 gDNA, this sequence is identical to the region between IS*Bma*1b and BTH_I2587 (pABT74) or the region between BTH_I2743 and IS*2*β (pABT73). The PCR product was then cloned into the blunt end of pJET1.2 followed by verification of the resulting plasmid. The sequence of interested was digested from the pJET1.2 backbone using BglII and then cloned into the BamHI restriction site of pEX18-TMP [65] giving rise to pABT73-TMP and pABT74-TMP. The plasmid was then moved to RHO3 cells for conjugation into *Bt*Reg1::*nptII* (pABT74-TMP) and strain 131–10 (pABT73-TMP), followed by selection on kanamycin- and TMP-supplemented media. At least two independent cointegrants obtained from each mating were used for plasmid rescue analyses.

### Re-sequencing of WT *B*.*thailandensis* E264

Genomic DNA was isolated from WT *Bt*E264 cells grown in liquid broth using Wizard Genomic DNA Purification Kit (Promega). Paired-end TruSeq (Illumina) gDNA libraries were generated and subjected to sequencing for 300 cycles using the Illumina MiSeq platform at the High-Throughput Sequencing Facility (HTSF) at the UNC School of Medicine. Following demultiplexing, FASTQ files were mapped to the reference genome available for *Bt*E264 (Accession no. CP000086.1 for Chromosome I and CP000085.1 for Chromosome II) using the Geneious v. 8 standard assembler, resulting in >200x coverage. Sequencing reads can be accessed in the Sequence Read Archive (SRA); accession number PRJNA510167.

### PCR analyses to detect the junction of IS*2* megacircles

Primers Circ1 and Circ2 (S3 Table) were designed to bind at each end of, and reading in opposite direction away from, the composite transposon. Upon formation of the IS*2* megacircle, Circ1 and Circ2 are in proximity and in the correct orientation to generate a product. At least 15 independent PCR products have been sequenced. To detect WT DNA, primers In1 and In2 (which bind to BTH_I2615 and BTH_I2616, respectively, to amplify a 1.0 kb fragment) were used. PCR studies were performed in 25 μL reaction mixtures with GoTaq DNA polymerase (Promega) for 25 cycles and with 3 μL of diluted over-night cultures normalized an OD_600_ of 1.0 as the source of template DNA. PCR conditions to detect the megacircle junction included an annealing temperature of 55°C and elongation time of 150 seconds. PCR products were analyzed on a 0.8% agarose gel containing GelRed Nucleic Acid Gel Stain (Biotium) and visualized under UV light.

### Colony biofilm interbacterial competitions

Competitions between inhibitors and Δ*bcpAIOB* (which is susceptible to killing via CDI due to the absence of *bcpI*) were performed as previously described [8]. Briefly, overnight liquid cultures of inhibitors and target were diluted to OD_600_ of 0.2, and single inhibitors were mixed with Δ*bcpAIOB* at a 1:1 ratio. Next, 20 μL of the cell mixture were spotted in triplicate onto LSLB agar without antibiotic selection. Plates were incubated at room temperature for exactly 24 hours. Bacteria from the edge of the colony biofilm were harvested and suspended in PBS, then subjected to serial dilutions and plated on LSLB supplemented with appropriate antibiotics to enumerate CFU corresponding to the inhibitor and target. The competitive index (C.I.) is reported as the log of the ratio of inhibitor to target cells at 24 hours (*t*_24_) divided by the same ratio at *t*_0_. Three biological replicates were performed for each competition.

### Community-associated phenotypes

Congo red (CR) binding was determined by counting the number of CR+ and CR- colonies from strains grown on M63 minimal medium supplemented with 40 μg/mL of Congo red dye. Upon inoculation, plates were cultured at 37°C for 48 hours then incubated at room temperature for approximately three days. The ability of WT and mutant strains to aggregate at the air-liquid interphase when grown in M63 minimal medium was determined as follows. Overnight LSLB cultures were washed with PBS and diluted to an OD_600_ of 0.2 with M63 supplemented with 0.01% casamino acids and 0.4% glycerol in a final volume of 2 mL. Bacteria were cultured at 37°C while rotating for 24 hours, then imaged. Colony biofilm pigmentation assays were conducted as follows. Overnight LSLB cultures were washed and diluted to an OD_600_ of 0.2 with PBS, 20 μL of cell suspension was then spotted onto LSLB agar and air dried. Plates were incubated at room temperature for 2–3 weeks prior to imaging.

### Plasmid rescue

Plasmid rescue was performed using genomic DNA from two independent *Bt*Reg1::*nptII*::pABT74-TMP cointegrants and one 131–10::pABT73-TMP cointegrant. Genomic DNA (2 μg) was digested with 100 U of NotI (for strain *Bt*Reg1::*nptII*+pABT74-TMP) or SacII (for 131–10::pABT73-TMP) at 37°C for 18 hours, the reaction was then supplemented with additional 20 U of the restriction enzyme and incubated for two more hours. Next, the reaction was heat inactivated following manufactures’ recommendations. T4 ligase was added, and the reaction containing digested gDNA and ligase was incubated overnight at 16°C, then transformed into 5-alpha F’*I*^*q*^ High Efficiency Competent *E*. *coli* cells (NEB, C2992H), and transformants were selected on media supplemented with TMP. Lastly, “rescued” plasmids from multiple transformants were isolated and subjected to Sanger sequencing.

### Transformation efficiency

Natural transformation of *Bt*E264 was used with modifications [63]. Bacteria grown overnight in LSLB were used to inoculate M63 minimal medium at a 1:20 dilution, then incubated at 37°C for 5 hours. Cultures were concentrated to an OD_600_ of 10 (in M63 medium) and 50 μL of cell suspension were incubated at room temperature for 30 minutes with 100 ng of linearized plasmids (pABT63, pABT77 or pABT79) or 2 μL of water. Next, 1.5 mL of fresh M63 were added to each sample and transferred to a tube to be cultured at 37°C while rotating for 20 hours. Cells were then pelleted and resuspended in 40 μL of PBS, 20 μL were plated on LSLB supplemented with kanamycin. The remaining 20 μL were subjected to serial dilutions, which were then plated on LSLB without antibiotics. Plates were incubated at 37°C for 24 hours, after which CFU were counted. Transformation efficiency was calculated by dividing the number of kanamycin resistant colonies by the number of colonies on the LSLB plates without antibiotics. Limit of detection in LSLB media supplemented with kanamycin is equal to 2.

## Supporting information

S1 Fig Protein alignments using the orfAB gene product from BtE264 and E. coli. (**A**) The predicted amino acid sequence of IS*2* OrfAB from *Bt*E264 was aligned to the first identified OrfAB from *E*. *coli* K-12 (Accession number M18426, [28]) using the ClustalW multiple sequence alignment tool. There is 62.3% identity and 82.6% similarity between the two amino acid sequences. Residues boxed in red and green are the predicted DNA binding domain and the predicted catalytic site, respectively. (**B**) The predicted fusion protein OrfAB from *Bt*E264 aligned to the IS*26* transposase from *E*. *coli*. There is 10.8% identity and 33.1% similarity between the two amino acid sequences. Fully conserved residues are shaded in black; residues with similar properties are shaded in grey.
(TIF)

S2 Fig IS2-like elements in BtE264. A total of six IS*2*-like elements are found in the reference sequence of *Bt*E264. Each *orfAB* gene pair received a name, IS*2*α, IS*2*β, or IS*2*β 2–5. A 5 bp target repeat flanking each element was identified as well (red box). The 3’ ends of the inverted repeats are highlighted in yellow or orange.
(TIF)

S3 Fig PCR analyses to detect WT bcpAIOB or bcpAIOB::nptII. Strain Reg1::*nptII* is a merodiploid with two copies of *bcpAIOB*. Primers P11 and P12 amplify the 3’ end of *bcpA*. Replacement of *bcpAIOB* with *nptII* is confirmed with primers P13 (binds outside of the homology sequence used to introduce the mutation) and kan1 (binds to the *nptII* cassette).
(TIF)

S4 Fig Graphical Representation of binding site for primers used to detect WT DNA. (TIF)

S5 Fig Translocation of the mobile genetic did not disrupt CDI or CDS. (**A**) PCR analyses to detect the megacircle junction in the 131–10 strain. (**B**) CDI-mediated competitions between WT or the 131–10 mutant and *bcpAIOB*. The differences in C.I. values are not significant. (**C**) Intracellular mobilization of *bcpAIOB*-containing MGE did not have an effect on community-associated behaviors such as aggregation in M63 minimal medium, pigment production, or binding of Congo red dye. *P* values were obtained using Mann-Whitney U test comparing mutant strains to WT. Results for Congo red binding are shown as mean +/- SEM of three independent experiments (*n* = 6). *P < 0.05.
(TIF)

S6 Fig PCR analyses of additional IS*2*β elements present in BtE264. Detection of a PCR product of predicted size with the primers indicated confirms the sequences adjacent to the IS*2*β elements match the reference sequence.
(TIF)

S7 Fig Comparison of the “α end” of the composite transposon in BtE264 and corresponding region in BtE254. The 10 bps direct repeats present in *Bt*E264 are in red. Putative inverted repeats (IR) of the IS*Bma*1-containing element are shown as brown triangles. Homology between the strains is marked in yellow.
(TIF)

S1 Table Predicted orfs from the 210kb region with increased number of mapped reads. (DOC)

S2 Table Strains and plasmids used in this study. (DOC)

S3 Table Primers used in this study. (DOC)

S1 Dataset Excel spreadsheet containing underlying data for Figs 5 and 6 and S5 Fig. (XLSX)

## References

[1] Hansen SK, Rainey PB, Haagensen JAJ, Molin S. Evolution of species interactions in a biofilm community. Nature. 2007 2 1;445(7127):533–6. 10.1038/nature05514 17268468

[2] Ren D, Madsen JS, Sørensen SJ, Burmølle M. High prevalence of biofilm synergy among bacterial soil isolates in cocultures indicates bacterial interspecific cooperation. ISME J. 2015 Jan;9(1):81–9. 10.1038/ismej.2014.96 24936766 PMC4274433

[3] Rendueles O, Ghigo J-M. Mechanisms of Competition in Biofilm Communities. Microbiol Spectr. 2015 6;3(3). 10.1128/microbiolspec.MB-0009-2014 26185066

[4] Flemming H-C, Wingender J, Szewzyk U, Steinberg P, Rice SA, Kjelleberg S. Biofilms: an emergent form of bacterial life. Nat Rev Microbiol. Nature Publishing Group; 2016 9 1;14(9):563–75. 10.1038/nrmicro.2016.94 27510863

[5] Foster KR, Bell T. Competition, not cooperation, dominates interactions among culturable microbial species. Curr Biol. 2012 10 9;22(19):1845–50. 10.1016/j.cub.2012.08.005 22959348

[6] Aoki SK, Pamma R, Hernday AD, Bickham JE, Braaten BA, Low DA. Contact-dependent inhibition of growth in *Escherichia coli*. Science. American Association for the Advancement of Science; 2005 8 19;309(5738):1245–8. 10.1126/science.1115109 16109881

[7] Mazar J, Cotter PA. New insight into the molecular mechanisms of two-partner secretion. Trends Microbiol. 2007 11;15(11):508–15. 10.1016/j.tim.2007.10.005 17988872

[8] Anderson MS, Garcia EC, Cotter PA. The Burkholderia bcpAIOB genes define unique classes of two-partner secretion and contact dependent growth inhibition systems. PLoS Genet. 2012;8(8):e1002877 10.1371/journal.pgen.1002877 22912595 PMC3415462

[9] Aoki SK, Diner EJ, de Roodenbeke CT, Burgess BR, Poole SJ, Braaten BA, et al A widespread family of polymorphic contact-dependent toxin delivery systems in bacteria. Nature. Nature Publishing Group; 2010 11 18;468(7322):439–42. 10.1038/nature09490 21085179 PMC3058911

[10] Nikolakakis K, Amber S, Wilbur JS, Diner EJ, Aoki SK, Poole SJ, et al The toxin/immunity network of *Burkholderia pseudomallei* contact-dependent growth inhibition (CDI) systems. Mol Microbiol. Blackwell Publishing Ltd; 2012 5;84(3):516–29. 10.1111/j.1365-2958.2012.08039.x 22435733 PMC3331888

[11] Johnson PM, Gucinski GC, Garza-Sánchez F, Wong T, Hung L-W, Hayes CS, et al Functional Diversity of Cytotoxic tRNase/Immunity Protein Complexes from *Burkholderia pseudomallei*. J Biol Chem. 2016 9 9;291(37):19387–400. 10.1074/jbc.M116.736074 27445337 PMC5016678

[12] Morse RP, Nikolakakis KC, Willett JLE, Gerrick E, Low DA, Hayes CS, et al Structural basis of toxicity and immunity in contact-dependent growth inhibition (CDI) systems. Proc Natl Acad Sci USA. National Acad Sciences; 2012 12 26;109(52):21480–5. 10.1073/pnas.1216238110 23236156 PMC3535622

[13] Dawkins R. The Selfish Gene. Oxford University Press, USA; 1976. 1 p.

[14] Danka ES, Garcia EC, Cotter PA. Are CDI Systems Multicolored, Facultative, Helping Greenbeards? Trends Microbiol. 2017 5;25(5):391–401. 10.1016/j.tim.2017.02.008 28285908 PMC5400692

[15] West SA, Gardner A. Altruism, Spite, and Greenbeards. Science. American Association for the Advancement of Science; 2010 3 12;327(5971):1341–4. 10.1126/science.1178332 20223978

[16] Gardner A, West SA. Greenbeards. Evolution. Wiley/Blackwell (10.1111); 2010 1 1;64(1):25–38. 10.1111/j.1558-5646.2009.00842.x 19780812

[17] Aoki SK, Malinverni JC, Jacoby K, Thomas B, Pamma R, Trinh BN, et al Contact-dependent growth inhibition requires the essential outer membrane protein BamA (YaeT) as the receptor and the inner membrane transport protein AcrB. Mol Microbiol. Blackwell Publishing Ltd; 2008 10;70(2):323–40. 10.1111/j.1365-2958.2008.06404.x 18761695 PMC2579741

[18] Ruhe ZC, Low DA, Hayes CS. Bacterial contact-dependent growth inhibition. Trends Microbiol. 2013 5;21(5):230–7. 10.1016/j.tim.2013.02.003 23473845 PMC3648609

[19] Willett JLE, Gucinski GC, Fatherree JP, Low DA, Hayes CS. Contact-dependent growth inhibition toxins exploit multiple independent cell-entry pathways. Proc Natl Acad Sci USA. National Acad Sciences; 2015 9 8;112(36):11341–6. 10.1073/pnas.1512124112 26305955 PMC4568652

[20] Beck CM, Willett JLE, Cunningham DA, Kim JJ, Low DA, Hayes CS. CdiA Effectors from Uropathogenic Escherichia coli Use Heterotrimeric Osmoporins as Receptors to Recognize Target Bacteria. Baumler AJ, editor. PLoS Pathog. 2016 10;12(10):e1005925 10.1371/journal.ppat.1005925 27723824 PMC5056734

[21] Diner EJ, Beck CM, Webb JS, Low DA, Hayes CS. Identification of a target cell permissive factor required for contact-dependent growth inhibition (CDI). Genes Dev. Cold Spring Harbor Lab; 2012 3 1;26(5):515–25. 10.1101/gad.182345.111 22333533 PMC3305988

[22] Kaundal S, Uttam M, Thakur KG. Dual Role of a Biosynthetic Enzyme, CysK, in Contact Dependent Growth Inhibition in Bacteria. Yin HH, editor. PLoS ONE. 2016;11(7):e0159844 10.1371/journal.pone.0159844 27458806 PMC4961446

[23] Jones AM, Garza-Sánchez F, So J, Hayes CS, Low DA. Activation of contact-dependent antibacterial tRNase toxins by translation elongation factors. Proc Natl Acad Sci USA. 2017 3 7;114(10):E1951–7. 10.1073/pnas.1619273114 28223500 PMC5347540

[24] Anderson MS, Garcia EC, Cotter PA. Kind discrimination and competitive exclusion mediated by contact-dependent growth inhibition systems shape biofilm community structure. PLoS Pathog. 2014 4;10(4):e1004076 10.1371/journal.ppat.1004076 24743836 PMC3990724

[25] Garcia EC, Anderson MS, Hagar JA, Cotter PA. *Burkholderia* BcpA mediates biofilm formation independently of interbacterial contact-dependent growth inhibition. Mol Microbiol. 2013 9;89(6):1213–25. 10.1111/mmi.12339 23879629 PMC3786370

[26] Garcia EC, Perault AI, Marlatt SA, Cotter PA. Interbacterial signaling via *Burkholderia* contact-dependent growth inhibition system proteins. Proc Natl Acad Sci USA. National Acad Sciences; 2016 7 19;113(29):8296–301. 10.1073/pnas.1606323113 27335458 PMC4961174

[27] Hirsch H-J, Starlinger P, Brachet P. Two kinds of insertions in bacterial genes. Mol Gen Genet. Springer-Verlag; 1972;119(3):191–206. 4567154 10.1007/BF00333858

[28] Ghosal D, Sommer H, Saedler H. Nucleotide sequence of the transposable DNA-element IS*2*. Nucleic Acids Res. 1979 3;6(3):1111–22. 375194 10.1093/nar/6.3.1111 PMC327757

[29] Ronecker H-J, Rak B. Genetic organization of insertion element IS*2* based on a revised nucleotide sequence. Gene. 1987 1;59(2–3):291–6. 2830172 10.1016/0378-1119(87)90337-4

[30] Hu ST, Hwang JH, Lee LC, Lee CH, Li PL, Hsieh YC. Functional analysis of the 14 kDa protein of insertion sequence *2*. J Mol Biol. 1994 2 18;236(2):503–13. 10.1006/jmbi.1994.1161 8107136

[31] Lewis LA, Grindley ND. Two abundant intramolecular transposition products, resulting from reactions initiated at a single end, suggest that IS*2* transposes by an unconventional pathway. Mol Microbiol. 1997 8;25(3):517–29. 9302014 10.1046/j.1365-2958.1997.4871848.x

[32] Hu ST, Lee LC, Lei GS. Detection of an IS*2*-encoded 46-kilodalton protein capable of binding terminal repeats of IS*2*. J Bacteriol. American Society for Microbiology (ASM); 1996 10;178(19):5652–9. 8824609 10.1128/jb.178.19.5652-5659.1996 PMC178403

[33] Siguier P, Gourbeyre E, Varani A, Ton Hoang B, Chandler M. Everyman's Guide to Bacterial Insertion Sequences. Microbiol Spectr. 2015 4;3(2):MDNA3–0030–2014. 10.1128/microbiolspec.MDNA3-0030-2014 26104715

[34] Sekine Y, Eisaki N, Ohtsubo E. Translational control in production of transposase and in transposition of insertion sequence IS*3*. J Mol Biol. 1994 2 4;235(5):1406–20. 10.1006/jmbi.1994.1097 8107082

[35] Ichikawa H, Ikeda K, Wishart WL, Ohtsubo E. Specific binding of transposase to terminal inverted repeats of transposable element Tn3. Proceedings of the National Academy of Sciences. National Academy of Sciences; 1987 12 1;84(23):8220–4. 10.1073/pnas.84.23.8220 PMC299513 2825182

[36] Derbyshire KM, Hwang L, Grindley ND. Genetic analysis of the interaction of the insertion sequence IS*903* transposase with its terminal inverted repeats. Proceedings of the National Academy of Sciences. National Academy of Sciences; 1987 11 1;84(22):8049–53. 10.1073/pnas.84.22.8049 PMC299474 2825175

[37] Lewis LA, Gadura N, Greene M, Saby R, Grindley ND. The basis of asymmetry in IS*2* transposition. Mol Microbiol. 2001 11;42(4):887–901. 11737634 10.1046/j.1365-2958.2001.02662.x

[38] Nagy Z, Chandler M. Regulation of transposition in bacteria. Res Microbiol. 2004 6;155(5):387–98. Available from: 10.1016/j.resmic.2004.01.008 15207871

[39] Mollet B, Iida S, Shepherd J, Arber W. Nucleotide sequence of IS*26*, a new prokaryotic mobile genetic element. Nucleic Acids Res. Oxford University Press; 1983 9 24;11(18):6319–30. 6312419 10.1093/nar/11.18.6319 PMC326375

[40] Iida S, Mollet B, Meyer J, W A, 1984. Functional characterization of the prokaryotic mobile genetic element IS*26*. Mol Gen Genet. 1984;198(1):84–9. 6097800 10.1007/BF00328705

[41] Mollet B, Iida S, W A, 1985. Gene organization and target specificity of the prokaryotic mobile genetic element IS*26*. Mol Gen Genet. 1985;201(2):198–203. 3003524 10.1007/BF00425660

[42] Harmer CJ, Hall RM. Targeted conservative formation of cointegrates between two DNA molecules containing IS*26* occurs via strand exchange at either IS end. Mol Microbiol. 2017 8 23;237:301. 10.1111/mmi.13774 28833671

[43] Harmer CJ, Hall RM. IS26-Mediated Precise Excision of the IS*26*-*aphA1a* Translocatable Unit. MBio. American Society for Microbiology; 2015 12 8;6(6):e01866–15. 10.1128/mBio.01866-15 26646012 PMC4676283

[44] Harmer CJ, Moran RA, Hall RM. Movement of IS*26*-associated antibiotic resistance genes occurs via a translocatable unit that includes a single IS*26* and preferentially inserts adjacent to another IS*26*. MBio. American Society for Microbiology; 2014 10 7;5(5):e01801–14. 10.1128/mBio.01801-14 25293759 PMC4196232

[45] Harmer CJ, Hall RM. IS26-Mediated Formation of Transposons Carrying Antibiotic Resistance Genes. mSphere. American Society for Microbiology Journals; 2016 3;1(2):e00038–16. 10.1128/mSphere.00038-16 27303727 PMC4894685

[46] Lewis LA, Astatke M, Umekubo PT, Alvi S, Saby R, Afrose J, et al Protein-DNA interactions define the mechanistic aspects of circle formation and insertion reactions in IS*2* transposition. Mob DNA. BioMed Central Ltd; 2012;3(1):1 10.1186/1759-8753-3-1 22277150 PMC3299598

[47] Lewis LA, Astatke M, Umekubo PT, Alvi S, Saby R, Afrose J. Soluble expression, purification and characterization of the full length IS*2* Transposase. Mob DNA. BioMed Central; 2011;2(1):14. 10.1186/1759-8753-2-14 PMC3219604 22032517

[48] Lewis LA, Cylin E, Lee HK, Saby R, Wong W, Grindley NDF. The left end of IS*2*: a compromise between transpositional activity and an essential promoter function that regulates the transposition pathway. J Bacteriol. 2004 2;186(3):858–65. 10.1128/JB.186.3.858-865.2004 14729714 PMC321474

[49] Duval-Valentin G, Chandler M. Cotranslational control of DNA transposition: a window of opportunity. Mol Cell. 2011 12 23;44(6):989–96. 10.1016/j.molcel.2011.09.027 22195971

[50] Gonçalves GAL, Oliveira PH, Gomes AG, Prather KLJ, Lewis LA, Prazeres DMF, et al Evidence that the insertion events of IS*2* transposition are biased towards abrupt compositional shifts in target DNA and modulated by a diverse set of culture parameters. Appl Microbiol Biotechnol. Springer Berlin Heidelberg; 2014 8;98(15):6609–19. 10.1007/s00253-014-5695-6 24769900

[51] Ford PJ, Avison MB. Evolutionary mapping of the SHV *β*-lactamase and evidence for two separate IS*26*-dependent *blaSHV* mobilization events from the *Klebsiella pneumoniae* chromosome. J Antimicrob Chemother. Oxford University Press; 2004 7 1;54(1):69–75. 10.1093/jac/dkh251 15163647

[52] Doublet B, Praud K, Weill F-X, Cloeckaert A. Association of IS*26*-composite transposons and complex In4-type integrons generates novel multidrug resistance loci in Salmonella genomic island 1. J Antimicrob Chemother. 2009 2;63(2):282–9. 10.1093/jac/dkn500 19074421

[53] Allard JD, Gibson ML, Vu LH, Nguyen TT, Bertrand KP. Nucleotide sequence of class D tetracycline resistance genes from *Salmonella ordonez*. Mol Gen Genet. Springer-Verlag; 1993 2;237(1–2):301–5. 8384294 10.1007/BF00282811

[54] Miriagou V, Carattoli A, Tzelepi E, Villa L, Tzouvelekis LS. IS*26*-Associated In4-Type Integrons Forming Multiresistance Loci in Enterobacterial Plasmids. Antimicrob Agents Chemother. American Society for Microbiology; 2005 8 1;49(8):3541–3. 10.1128/AAC.49.8.3541-3543.2005 16048979 PMC1196216

[55] Zienkiewicz M, Kern-Zdanowicz I, Carattoli A, Gniadkowski M, Cegłowski P. Tandem multiplication of the IS*26*-flanked amplicon with the *bla*(SHV-5) gene within plasmid p1658/97. FEMS Microbiol Lett. 2013 4;341(1):27–36. 10.1111/1574-6968.12084 23330672

[56] Gallagher LA, Ramage E, Patrapuvich R, Weiss E, Brittnacher M, Manoil C. Sequence-defined transposon mutant library of *Burkholderia thailandensis*. MBio. American Society for Microbiology; 2013;4(6):e00604–13. 10.1128/mBio.00604-13 24194535 PMC3870259

[57] Perault AI, Cotter PA, DiRita VJ. Three Distinct Contact-Dependent Growth Inhibition Systems Mediate Interbacterial Competition by the Cystic Fibrosis Pathogen *Burkholderia dolosa*. J Bacteriol. American Society for Microbiology Journals; 2018 11 15;200(22):e00428–18. 10.1128/JB.00428-18 PMC6199481 30150233

[58] Yu Y, Kim HS, Chua HH, Lin CH, Sim SH, Lin D, et al Genomic patterns of pathogen evolution revealed by comparison of *Burkholderia pseudomallei*, the causative agent of melioidosis, to avirulent *Burkholderia thailandensis*. BMC Microbiol. BioMed Central; 2006 5 26;6(1):46. 16725056 10.1186/1471-2180-6-46 PMC1508146

[59] Tuanyok A, Leadem BR, Auerbach RK, Beckstrom-Sternberg SM, Beckstrom-Sternberg JS, Mayo M, et al Genomic islands from five strains of *Burkholderia pseudomallei*. BMC Genomics. 2008 11 27;9(1):566. 19038032 10.1186/1471-2164-9-566 PMC2612704

[60] Brett PJ, DeShazer D, Woods DE. *Burkholderia thailandensis* sp. nov., a *Burkholderia pseudomallei*-like species. Int J Syst Bacteriol. 1998 1;48 Pt 1(1):317–20. 9542103 10.1099/00207713-48-1-317

[61] López CM, Rholl DA, Trunck LA, Schweizer HP. Versatile dual-technology system for markerless allele replacement in *Burkholderia pseudomallei*. Appl Environ Microbiol. American Society for Microbiology; 2009 10;75(20):6496–503. 10.1128/AEM.01669-09 19700544 PMC2765137

[62] Norris MH, Kang Y, Wilcox B, Hoang TT. Stable, site-specific fluorescent tagging constructs optimized for *Burkholderia* species. Appl Environ Microbiol. American Society for Microbiology; 2010 11;76(22):7635–40. 10.1128/AEM.01188-10 20851961 PMC2976199

[63] Thongdee M, Gallagher LA, Schell M, Dharakul T, Songsivilai S, Manoil C. Targeted mutagenesis of *Burkholderia thailandensis* and Burkholderia pseudomallei through natural transformation of PCR fragments. Appl Environ Microbiol. American Society for Microbiology; 2008 5;74(10):2985–9. 10.1128/AEM.00030-08 18310423 PMC2394929

[64] Choi K-H, Mima T, Casart Y, Rholl D, Kumar A, Beacham IR, et al Genetic tools for select-agent-compliant manipulation of *Burkholderia pseudomallei*. Appl Environ Microbiol. American Society for Microbiology; 2008 2;74(4):1064–75. 10.1128/AEM.02430-07 18156318 PMC2258562

[65] Barrett AR, Kang Y, Inamasu KS, Son MS, Vukovich JM, Hoang TT. Genetic tools for allelic replacement in *Burkholderia* species. Appl Environ Microbiol. American Society for Microbiology; 2008 7;74(14):4498–508. 10.1128/AEM.00531-08 18502918 PMC2493169

