# Supplementary figures and images for: CDI/CDS system-encoding genes of Burkholderia thailandensis are located in a mobile genetic element that defines a new class of transposon
Source: PLoS Genet. 2019 Jan 7;15(1):e1007883. doi: 10.1371/journal.pgen.1007883 (PMC6350997; doi:10.1371/journal.pgen.1007883)

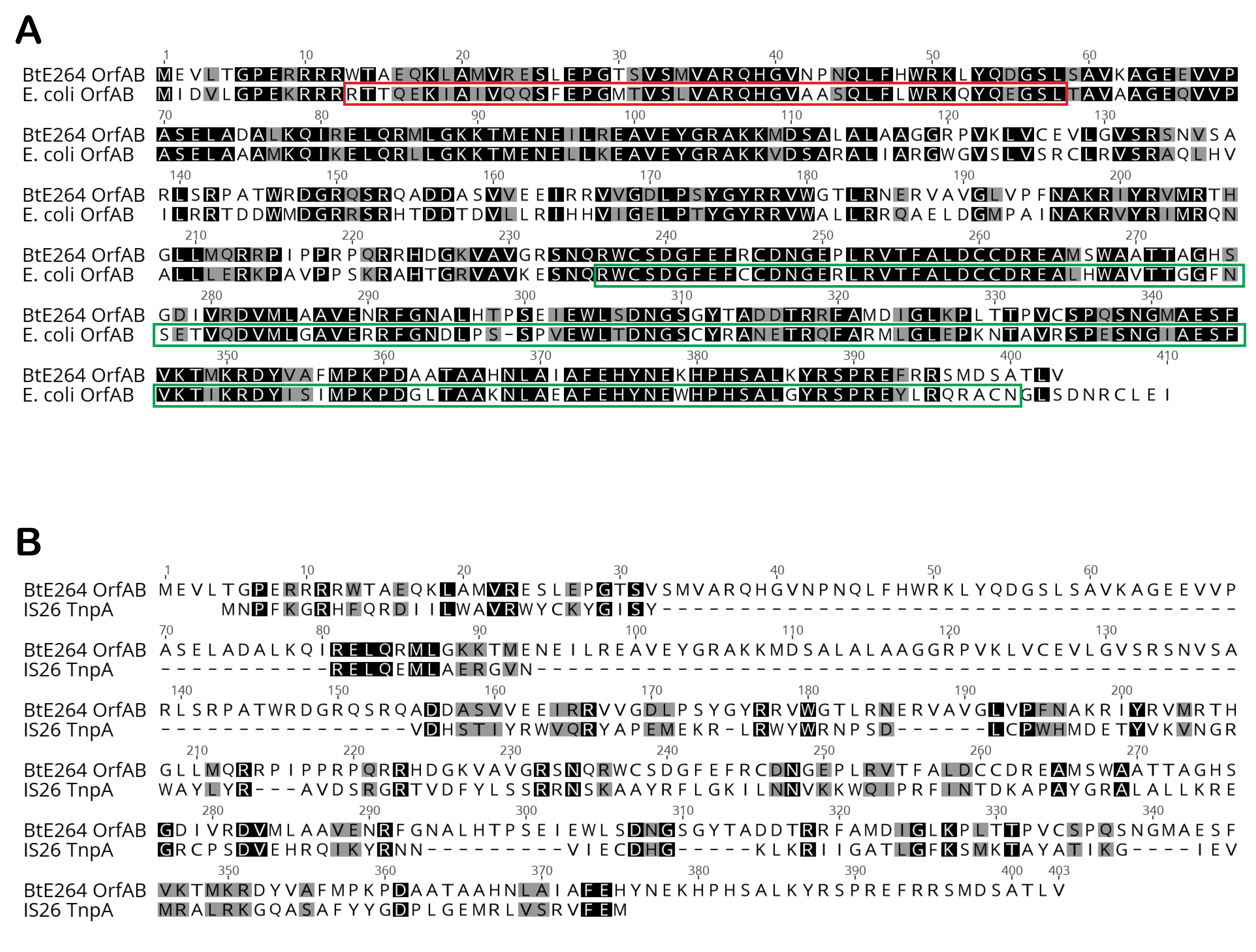

Supplement: S1 Fig — (A) The predicted amino acid sequence of IS2 OrfAB from BtE264 was aligned to the first identified OrfAB from E. coli K-12 (Accession number M18426, [28]) using the ClustalW multiple sequence alignment tool. There is 62.3% identity and 82.6% similarity between the two amino acid sequences. Residues boxed in red and green are the predicted DNA binding domain and the predicted catalytic site, respectively. (B) The predicted fusion protein OrfAB from BtE264 aligned to the IS26 transposase from E. coli. There is 10.8% identity and 33.1% similarity between the two amino acid sequences. Fully conserved residues are shaded in black; residues with similar properties are shaded in grey. (TIF) [file pgen.1007883.s001.tif]

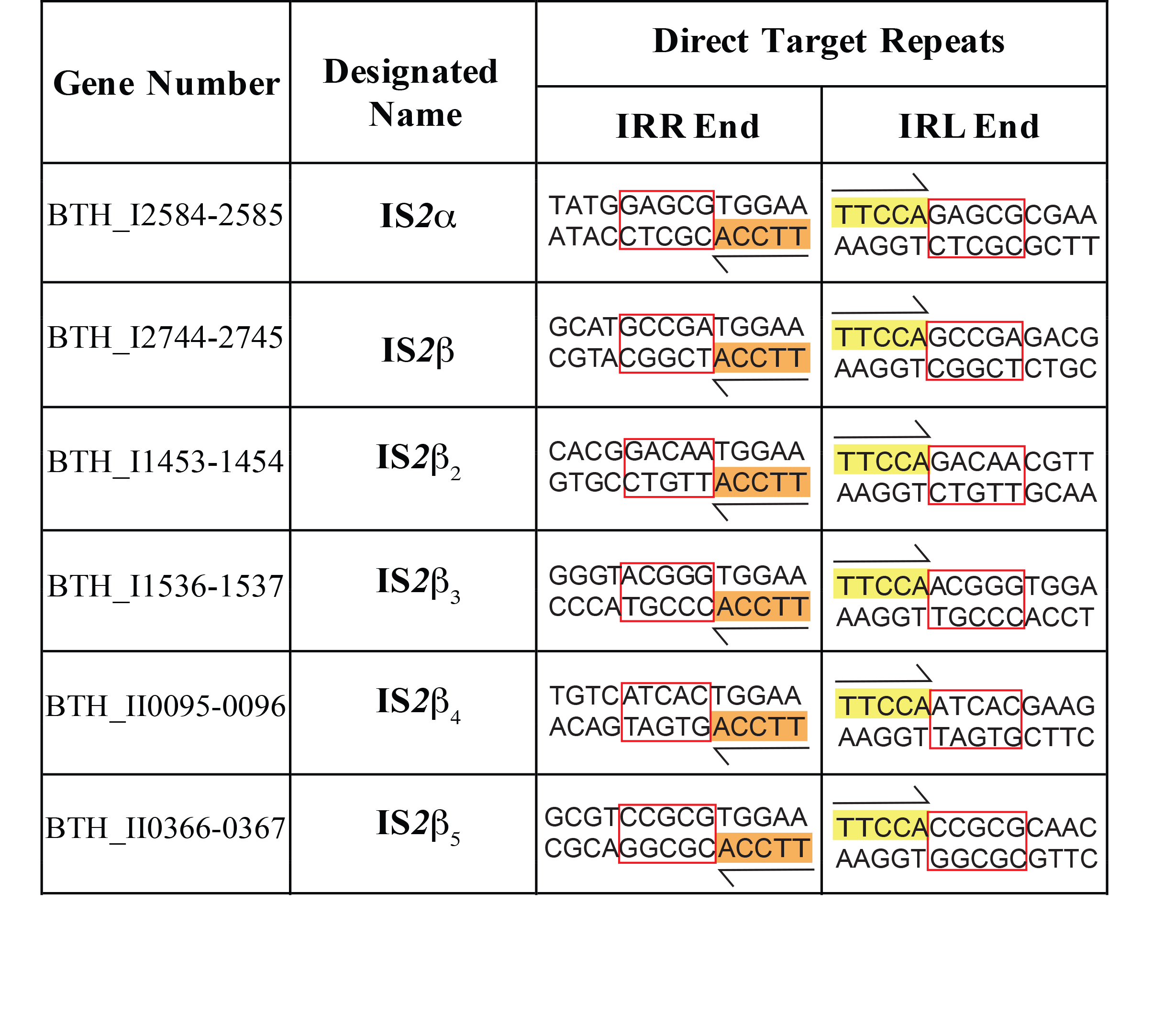

Supplement: S2 Fig — A total of six IS2-like elements are found in the reference sequence of BtE264. Each orfAB gene pair received a name, IS2α, IS2β, or IS2β 2–5. A 5 bp target repeat flanking each element was identified as well (red box). The 3’ ends of the inverted repeats are highlighted in yellow or orange. (TIF) [file pgen.1007883.s002.tif]

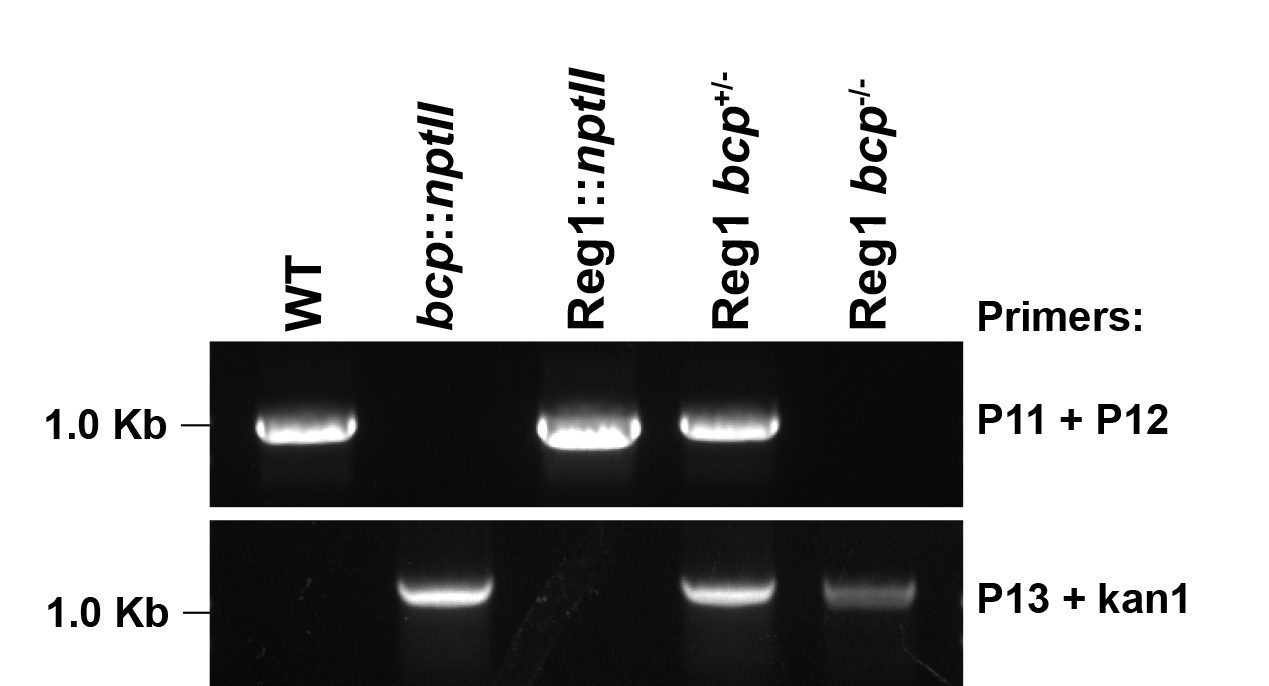

Supplement: S3 Fig — Strain Reg1::nptII is a merodiploid with two copies of bcpAIOB. Primers P11 and P12 amplify the 3’ end of bcpA. Replacement of bcpAIOB with nptII is confirmed with primers P13 (binds outside of the homology sequence used to introduce the mutation) and kan1 (binds to the nptII cassette). (TIF) [file pgen.1007883.s003.tif]

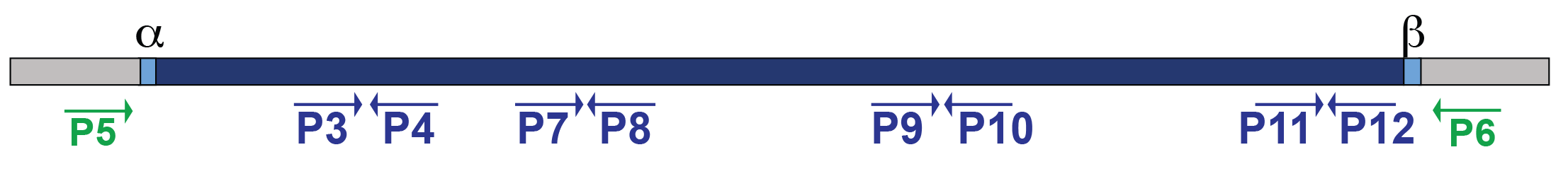

Supplement: S4 Fig — (TIF) [file pgen.1007883.s004.tif]

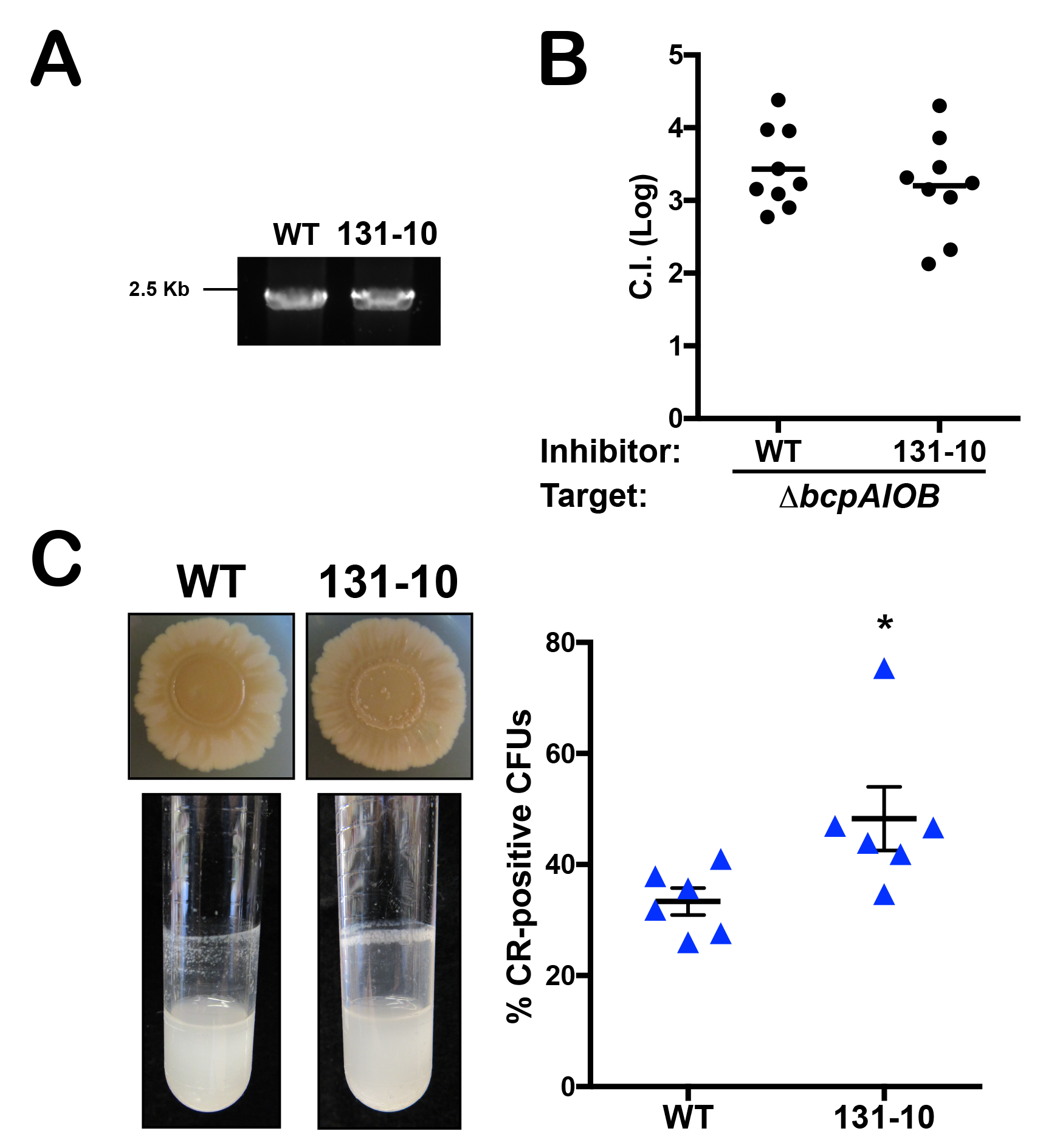

Supplement: S5 Fig — (A) PCR analyses to detect the megacircle junction in the 131–10 strain. (B) CDI-mediated competitions between WT or the 131–10 mutant and bcpAIOB. The differences in C.I. values are not significant. (C) Intracellular mobilization of bcpAIOB-containing MGE did not have an effect on community-associated behaviors such as aggregation in M63 minimal medium, pigment production, or binding of Congo red dye. P values were obtained using Mann-Whitney U test comparing mutant strains to WT. Results for Congo red binding are shown as mean +/- SEM of three independent experiments (n = 6). *P < 0.05. (TIF) [file pgen.1007883.s005.tif]

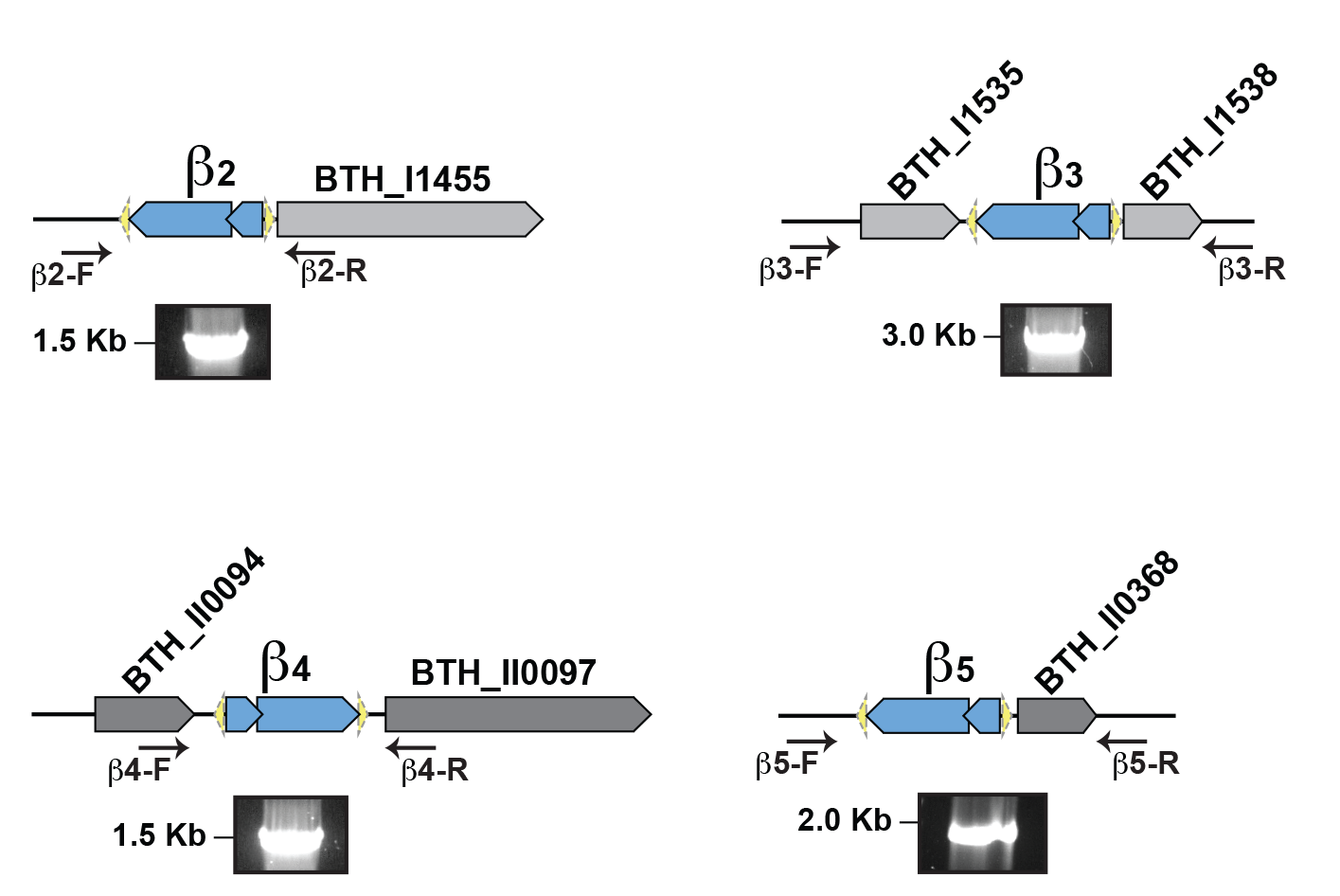

Supplement: S6 Fig — Detection of a PCR product of predicted size with the primers indicated confirms the sequences adjacent to the IS2β elements match the reference sequence. (TIF) [file pgen.1007883.s006.tif]

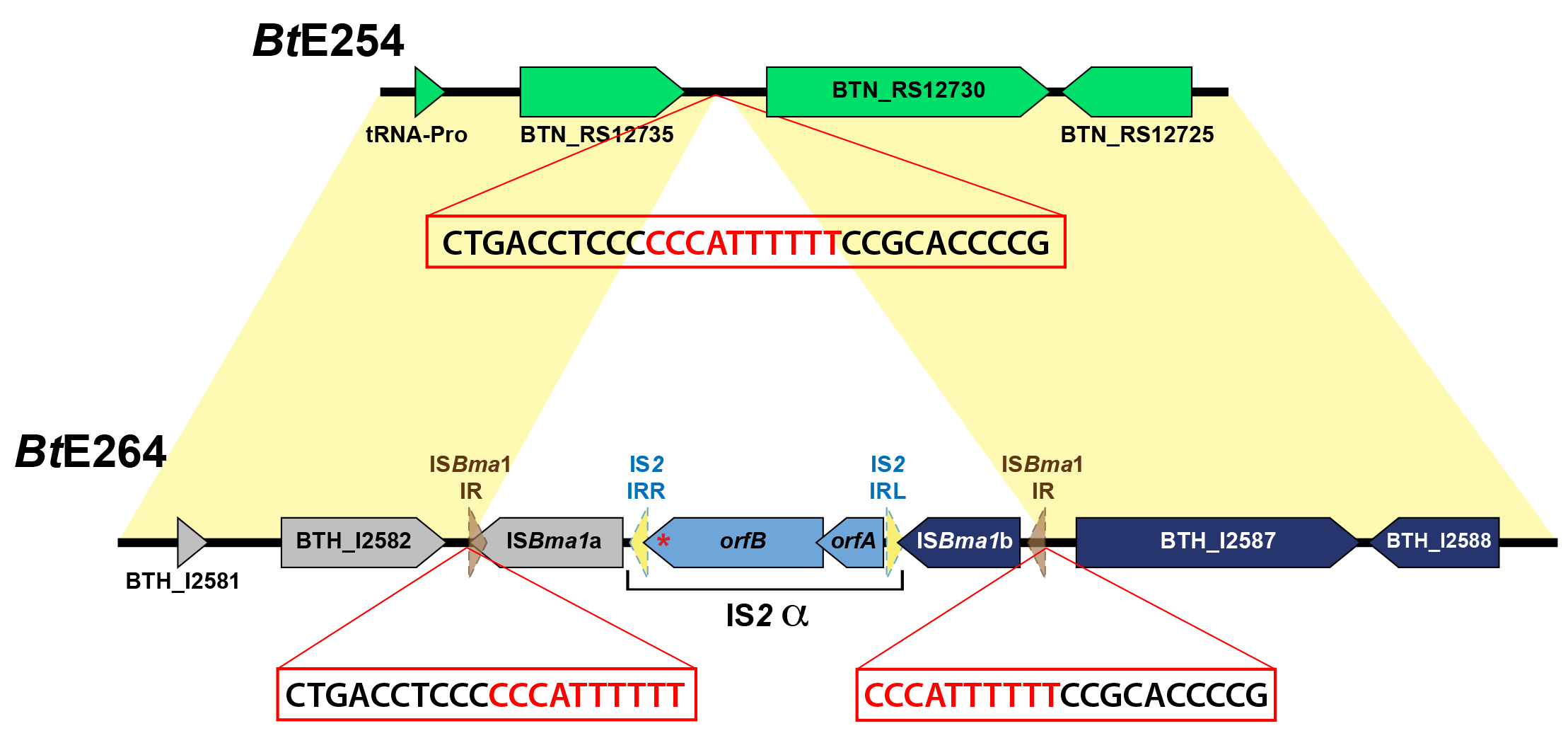

Supplement: S7 Fig — The 10 bps direct repeats present in BtE264 are in red. Putative inverted repeats (IR) of the ISBma1-containing element are shown as brown triangles. Homology between the strains is marked in yellow. (TIF) [file pgen.1007883.s007.tif]
