# Supplementary material for: CDI/CDS system-encoding genes of Burkholderia thailandensis are located in a mobile genetic element that defines a new class of transposon
Source: PLoS Genet. 2019 Jan 7;15(1):e1007883. doi: 10.1371/journal.pgen.1007883 (PMC6350997; doi:10.1371/journal.pgen.1007883)
Supplement: S1 Table — (DOC) [file pgen.1007883.s008.doc]

**Table S1. Predicted *orf*s from the 210kb region with increased number of mapped reads**

| **Locus tag** | **Gene** | **Direction** | **Product** |
| --- | --- | --- | --- |
| BTH_I2583 |  | reverse | IS*Bma1*a, transposase |
| BTH_I2584 |  | reverse | IS*2 orfB* |
| BTH_I2585 |  | reverse | IS*2 orfA* |
| BTH_I2586 |  | reverse | IS*Bma1*b, transposase |
| BTH_I2587 |  | forward | Predicted lipoprotein |
| BTH_I2588 |  | reverse | MerR family transcriptional regulator |
| BTH_I2589 | *ihfA* | reverse | Integration host factor, alpha subunit |
| BTH_I2590 | *pheT* | reverse | Phenylalanyl-tRNA synthetase, beta subunit |
| BTH_I2591 | *pheS* | reverse | Phenylalanyl-tRNA synthetase, alpha subunit |
| BTH_I2592 | *rplT* | reverse | 50S ribosomal protein L20 |
| BTH_I2593 | *rpmI* | reverse | 50S ribosomal protein L35 |
| BTH_I2594 | *infC* | reverse | Translation initiation factor IF-3 |
| BTH_I2595 | *thrS* | reverse | threonyl-tRNA synthetase |
| BTH_I2597 | *relA* | reverse | GTP pyrophosphokinase |
| BTH_I2598 |  | reverse | Endoribonuclease L-PSP superfamily |
| BTH_I2599 |  | reverse | Alpha/beta fold family hydrolase |
| BTH_I2600 |  | reverse | Hypothetical protein |
| BTH_I2601 |  | forward | Flavoprotein reductase |
| BTH_I2602 |  | forward | Hypothetical protein |
| BTH_I2603 |  | reverse | LysR family transcriptional regulator |
| BTH_I2604 |  | forward | Hypothetical protein |
| BTH_I2605 |  | forward | polysaccharide deacetylase family protein |
| BTH_I2606 |  | forward | Short chain dehydrogenase |
| BTH_I2607 | *scoB* | reverse | 3-oxoadipate CoA-succinyl transferase, beta subunit |
| BTH_I2608 | *scoA* | reverse | 3-oxoadipate CoA-succinyl transferase, alpha subunit |
| BTH_I2609 |  | forward | LuxR family transcriptional regulator |
| BTH_I2610 |  | forward | Hypothetical protein |
| BTH_I2611 |  | reverse | Thioesterase family protein |
| BTH_I2612 |  | reverse | Short chain dehydrogenase |
| BTH_I2613 |  | forward | Electron transfer flavoprotein-ubiquinone oxidoreductase |
| BTH_I2614 | *aroC* | reverse | Chorismate synthase |
| BTH_I2615 |  | reverse | Acyltransferase family protein |
| BTH_I2616 |  | reverse | CBS domain-containing protein |
| BTH_I2617 |  | forward | Hypothetical protein |
| BTH_I2618 |  | reverse | YihY family protein |
| BTH_I2619 |  | forward | Flavodoxin |
| BTH_I2620 |  | forward | Hypothetical protein |
| BTH_I2621 |  | forward | FAD-binding oxidoreductase |
| BTH_I2622 |  | forward | Ser/Thr protein phosphatase family protein |
| BTH_I2623 |  | reverse | Hypothetical protein |
| BTH_I2624 |  | reverse | Putative transcriptional regulator |
| BTH_I2625 |  | forward | NodT family RND efflux system outer membrane lipoprotein |
| BTH_I2626 |  | forward | Fusaric acid resistance domain-containing protein |
| BTH_I2627 |  | forward | Hypothetical protein |
| BTH_I2628 |  | forward | Putative HlyD family secretion protein |
| BTH_I2629 |  | reverse | Methyl-accepting chemotaxis protein |
| BTH_I2630 |  | forward | Fosmidomycin resistance protein |
| BTH_I2631 |  | reverse | 5'-methylthioadenosine/S-adenosylhomocysteine nucleosidase |
| BTH_I2632 | *uvrD* | reverse | DNA helicase II |
| BTH_I2633 | *valS* | forward | Valyl-tRNA synthetase |
| BTH_I2634 | *galU-2* | forward | UTP-glucose-1-phosphate uridylyltransferase |
| BTH_I2635 |  | reverse | Putative lipoprotein |
| BTH_I2636 |  | reverse | Putative sensor histidine kinase |
| BTH_I2637 |  | reverse | LysM domain-containing protein |
| BTH_I2638 |  | reverse | DNA-binding response regulator |
| BTH_I2639 |  | reverse | Hypothetical protein |
| BTH_I2640 |  | reverse | Hypothetical protein |
| BTH_I2641 |  | reverse | Myo-inositol dehydrogenase |
| BTH_I2642 |  | reverse | Myo-inositol dehydrogenase |
| BTH_I2643 |  | reverse | SIS domain-containing protein |
| BTH_I2644 |  | forward | Sugar ABC transporter, periplasmic sugar-binding protein |
| BTH_I2645 |  | forward | Sugar ABC transporter, permease protein |
| BTH_I2646 |  | forward | Sugar ABC transporter, ATP-binding protein |
| BTH_I2647 |  | forward | iolC protein |
| BTH_I2648 |  | forward | iolD protein |
| BTH_I2649 |  | forward | iolE protein |
| BTH_I2650 |  | forward | iolB protein |
| BTH_I2651 |  | reverse | Branched-chain amino acid ABC transporter, ATP-binding protein |
| BTH_I2652 |  | reverse | Branched-chain amino acid ABC transporter, permease |
| BTH_I2653 |  | reverse | Branched-chain amino acid ABC transporter, permease |
| BTH_I2654 |  | reverse | Thioesterase family protein |
| BTH_I2655 |  | reverse | Iron-containing alcohol dehydrogenase |
| BTH_I2656 |  | forward | Hypothetical protein |
| BTH_I2657 |  | reverse | MutT/NUDIX pyrophosphatase |
| BTH_I2658 |  | reverse | Putative protease signal peptide protein |
| BTH_I2659 |  | reverse | Major Facilitator Superfamily |
| BTH_I2660 |  | forward | LysR family transcriptional regulator |
| BTH_I2661 | *alaS* | reverse | Alanyl-tRNA synthetase |
| BTH_I2662 |  | forward | CAIB/BAIF family protein |
| BTH_I2663 |  | forward | Putative lipoprotein |
| BTH_I2664 |  | reverse | Hypothetical protein |
| BTH_I2665 | *glnS* | forward | Glutaminyl-tRNA synthetase |
| BTH_I2666 |  | reverse | NUDIX domain-containing protein |
| BTH_I2667 |  | reverse | conserved hypothetical protein |
| BTH_I2668 |  | reverse | Beta-N-Acetylglucosaminidase |
| BTH_I2669 |  | forward | Hypothetical protein |
| BTH_I2670 |  | reverse | Di-haem cytochrome C peroxidase family protein |
| BTH_I2671 |  | forward | Acid phosphatase AcpA |
| BTH_I2672 |  | forward | Trans-2-enoyl-CoA reductase |
| BTH_I2673 |  | reverse | Trans-aconitate methyltransferase |
| BTH_I2674 |  | reverse | DNA-binding response regulator |
| BTH_I2675 |  | reverse | Sensor histidine kinase/response regulator |
| BTH_I2676 |  | reverse | Spore Coat Protein U domain-containing protein |
| BTH_I2677 |  | reverse | Fimbrial usher protein |
| BTH_I2678 |  | reverse | Fimbrial assembly chaperone |
| BTH_I2679 |  | reverse | Spore Coat Protein U domain-containing protein |
| BTH_I2680 |  | reverse | Hypothetical protein |
| BTH_I2681 |  | reverse | Hypothetical protein |
| BTH_I2682 |  | forward | Hypothetical protein |
| BTH_I2683 |  | reverse | LysR family transcriptional regulator |
| BTH_I2684 |  | forward | Major facilitator family transporter |
| BTH_I2685 |  | forward | Hydrolase |
| BTH_I2686 |  | forward | H-NS histone family protein |
| BTH_I2687 | *mntH* | reverse | Manganese/iron transporter |
| BTH_I2688 |  | reverse | TnpC protein |
| BTH_I2689 |  | reverse | TnpB protein |
| BTH_I2690 |  | reverse | Hypothetical protein |
| BTH_I2691 |  | reverse | Hypothetical protein |
| BTH_I2692 |  | reverse | Hypothetical protein |
| BTH_I2693 |  | reverse | Rhs element Vgr protein |
| BTH_I2694 |  | reverse | Hypothetical protein |
| BTH_I2695 |  | reverse | Hypothetical protein |
| BTH_I2696 |  | reverse | Hypothetical protein |
| BTH_I2697 |  | reverse | Rhs element Vgr protein |
| BTH_I2698 |  | reverse | Hypothetical protein |
| BTH_I2699 |  | reverse | Hypothetical protein |
| BTH_I2700 |  | reverse | Putative lipoprotein |
| BTH_I2701 |  | reverse | Hypothetical protein |
| BTH_I2702 |  | reverse | Putative lipoprotein |
| BTH_I2703 |  | reverse | Hypothetical protein |
| BTH_I2704 |  | reverse | Hypothetical protein |
| BTH_I2705 |  | reverse | Rhs element Vgr protein |
| BTH_I2706 |  | forward | PAAR motif-containin protein |
| BTH_I2707 |  | forward | Hypothetical protein |
| BTH_I2708 |  | reverse | Hypothetical protein |
| BTH_I2709 |  |  | Pseudogene |
| BTH_I2710 |  | reverse | Hypothetical protein |
| BTH_I2711 |  | forward | Hypothetical protein |
| BTH_I2713 | *phaZ* | forward | Poly(3-hydroxybutyrate) depolymerase |
| BTH_I2714 |  | reverse | DNA-binding protein BprA |
| BTH_I2715 |  | reverse | Major facilitator family transporter |
| BTH_I2716 |  | reverse | LysR family transcriptional regulator |
| BTH_I2717 |  | forward | Phage integrase |
| BTH_I2718 |  | reverse | Acetyltransferase |
| BTH_I2719 |  | reverse | Pathogenesis-related protein |
| BTH_I2720 |  | reverse | Hypothetical protein |
| BTH_I2721 | *bcpB* | reverse | CDI/CDS system outer membrane transporter |
|  | *bcpO* | reverse | CDI/CDS system protein |
| BTH_I2722 | *bcpI* | reverse | CDI/CDS system immunity protein |
| BTH_I2723 | *bcpA* | reverse | CDI/CDS system exotoxin |
| BTH_I2724 |  | forward | Hypothetical protein |
| BTH_I2725 |  | forward | Hypothetical protein |
| BTH_I2726 |  | forward | Hypothetical protein |
| BTH_I2727 |  | forward | Hypothetical protein |
| BTH_I2728 |  | forward | Hypothetical protein |
| BTH_I2729 |  | forward | Hypothetical protein |
| BTH_I2730 |  | forward | Plasmid related protein |
| BTH_I2731 |  | reverse | DNA-binding protein |
| BTH_I2732 |  | forward | Hypothetical protein |
| BTH_I2733 |  | reverse | Helix-turn-helix domain protein, putative |
| BTH_I2734 |  | forward | Predicted lipoprotein |
| BTH_I2735 |  | reverse | TnpC protein |
| BTH_I2736 |  | reverse | TnpB protein |
| BTH_I2737 |  | reverse | Hypothetical protein |
| BTH_I2738 |  | reverse | Hypothetical protein |
| BTH_I2739 |  | reverse | Putative DNA mismatch repair protein |
| BTH_I2740 |  | reverse | Type I restriction-modification system endonuclease |
| BTH_I2741 |  | reverse | Hypothetical protein |
| BTH_I2742 |  | reverse | Type I restriction-modification system specificity determinant |
| BTH_I2743 |  | reverse | Type I restriction system adenine methylase |
| BTH_I2744 |  | reverse | IS*2 orfB* |
| BTH_I2745 |  | reverse | IS*2 orfA* |
