# Supplementary material for: CDI/CDS system-encoding genes of Burkholderia thailandensis are located in a mobile genetic element that defines a new class of transposon
Source: PLoS Genet. 2019 Jan 7;15(1):e1007883. doi: 10.1371/journal.pgen.1007883 (PMC6350997; doi:10.1371/journal.pgen.1007883)
Supplement: S2 Table — (DOC) [file pgen.1007883.s009.doc]

**Table S2. Strains and Plasmids used in this study**

| **Strain** | **Lab ID** | **Description** | **Reference** |
| --- | --- | --- | --- |
| *E. coli* strains |  |  |  |
| DH5α |  | *E. coli,* F^-^φ80*lacZ*ΔM15 Δ*(lacZYA-argF)U169 deoR recA1 endA1 hsdR17(*r_K_^-^ m_K_^+^ *) phoA supE44* |  |
| RHO3 |  | SM10(λ*pir*) Δ*asd*::*FRT* Δ*aphA*::*FRT* | [1] |
| NEB 5-alpha |  | F’ *I^q^* (High Efficiency) | NEB |
|  |  |  |  |
| *B. thailandensis* strains |  |  |  |
| *Bt*E264 | 104-01 | Wild-type *B. thailandensis* E264 | [2] |
| IS*2*α*::nptII* | 132-18 | BTH_I2584 and I2585 replaced with *FRT*-*nptII*-*FRT* | This study |
| IS*2*β*::nptII* | 130-47 | BTH_I2744 and I2745 replaced with *FRT*-*nptII*-*FRT* | This study |
| ΔIS*2*β | 132-11 | *nptII* flipped out of 130-47; Kan^S^ | This study |
| *bcpA*_EKAA_ | 129-63 | *Bt*E264 with E3064A and K3066A substitutions in *bcpA* | [3] |
| Δ*bcpAIOB* | 132-39 | Lacks CDI/CDS encoding genes | [4] |
| *Bt*-*Bp* chimera | 107-57 | Conserved region of *Bt*E264 BcpA fused to the C-term of *Bp*1106a-1 | [5] |
| Reg1*::nptII* | 130-41 | BTH_I2587-I2630 replaced with *FRT*-*nptII*-*FRT*; Kan^R^ | This study |
| Reg1 Kan^S^ | 130-52 | *nptII* flipped out of 130-41 | This study |
| Reg1Reg3*::nptII* | 130-61 | BTH_I2671-I2705 replaced with *FRT*-*nptII*-*FRT* in strain 130-41; Kan^R^ | This study |
| Reg1Reg3 Kan^S^ | 130-63 | *nptII* flipped out of 130-61 | This study |
| Reg1Reg3-IS*2*β*::nptII* | 130-79 | BTH_I2706-I2745 replaced with *FRT*-*nptII*-*FRT* in strain 130-63; Kan^R^ | This study |
| Reg1Reg3-IS*2*β Kan^S^ | 131-03 | *nptII* flipped out of 130-79 | This study |
| 131-10 | 131-10 | Replaced MGE from its native location with *FRT*-*nptII*-*FRT* in strain 131-03; Kan^R^ | This study |
| Reg1*::nptII* ::pABT74-TMP | 131-51 | 130-41 with pABT74-TMP integrated upstream of IS*Bma*1b in the mobilized element; TMP^R^ | This study |
| 131-10::pABT73-TMP | 131-34 | 131-10 with pABT73-Tp integrated upstream of IS*2*β in the mobilized element; TMP^R^ | This study |
| Reg1 *bcp*^+/–^ (Kan^R^) | 131-12 | 130-52, merodiploid with *bcpAIOB*::*nptII* and WT *bcpAIOB* | This study |
| Reg1 *bcp*^+/–^ (Kan^S^) | 131-65 | *nptII* flipped out of 131-12 | This study |
| Reg1 *bcp*^–/–^ (Kan^R^) | 131-17 | 130-52, merodiploid with both *bcpAIOB* copies removed | This study |
|  |  |  |  |
|  |  |  |  |
|  |  |  |  |
| **Plasmid** | **Backbone** | **Description** | **Reference** |
| pJET1.2 |  | Amp^R^ | Invitrogen |
| pEX18-TMP |  | *Burkholderia* allelic exchange vector | [6] |
| pABT62 | pJET1.2 | Contains *nptII* coding sequence and promoter flanked by FRT sites and NdeI or EcoRV restriction sites | This study |
| pABT63 | pJET1.2 | Reg1::*nptII*; Region 1 encompasses BTH_I2587-I2630 | This study |
| pABT65 | pJET1.2 | Reg3::*nptII*; Region 3 encompasses BTH_I2671-I2705 | This study |
| pABT66 | pJET1.2 | IS*2*β::*FRT*-*nptII*-*FRT* | This study |
| pABT68 | pJET1.2 | Reg4*::nptII*; Region 4 encompasses BTH_I2706-I2743. This plasmid also removes IS*2*β | This study |
| pABT71 | pJET1.2 | Reg1-4::*nptII*; replaces nucleotides 2,945,740 to 3,156,451 with *FRT*-*nptII*-*FRT* | This study |
| pABT73-TMP | pEX18-TMP | To integrate TMP-containing suicide plasmid at the “β end” of the MGE | This study |
| pABT74-TMP | pEX18-TMP | To integrate TMP-containing suicide plasmid at the “α end” of the MGE | This study |
| pABT77 | pJET1.2 | To integrate *nptII* immediately upstream of BTH_I2578 | This study |
| pABT78 | pJET1.2 | IS*2*α::*FRT*-*nptII*-*FRT* | This study |
| pABT79 | pJET1.2 | To integrate *nptII* immediately upstream of BTH_I2615 | This study |

1. López CM, Rholl DA, Trunck LA, Schweizer HP. Versatile dual-technology system for markerless allele replacement in *Burkholderia pseudomallei*. Appl Environ Microbiol. American Society for Microbiology; 2009;75: 6496–6503. doi:10.1128/AEM.01669-09

2. Brett PJ, DeShazer D, Woods DE. *Burkholderia thailandensis* sp. nov., a *Burkholderia pseudomallei*-like species. Int J Syst Bacteriol. 1998;48 Pt 1: 317–320. doi:10.1099/00207713-48-1-317

3. Garcia EC, Anderson MS, Hagar JA, Cotter PA. *Burkholderia* BcpA mediates biofilm formation independently of interbacterial contact-dependent growth inhibition. Mol Microbiol. 2013;89: 1213–1225. doi:10.1111/mmi.12339

4. Anderson MS, Garcia EC, Cotter PA. The *Burkholderia bcpAIOB* genes define unique classes of two-partner secretion and contact dependent growth inhibition systems. PLoS Genet. 2012;8: e1002877. doi:10.1371/journal.pgen.1002877

5. Anderson MS, Garcia EC, Cotter PA. Kind discrimination and competitive exclusion mediated by contact-dependent growth inhibition systems shape biofilm community structure. PLoS Pathog. 2014;10: e1004076. doi:10.1371/journal.ppat.1004076

6. Barrett AR, Kang Y, Inamasu KS, Son MS, Vukovich JM, Hoang TT. Genetic tools for allelic replacement in *Burkholderia* species. Appl Environ Microbiol. American Society for Microbiology; 2008;74: 4498–4508. doi:10.1128/AEM.00531-08
