# Supplementary material for: CDI/CDS system-encoding genes of Burkholderia thailandensis are located in a mobile genetic element that defines a new class of transposon
Source: PLoS Genet. 2019 Jan 7;15(1):e1007883. doi: 10.1371/journal.pgen.1007883 (PMC6350997; doi:10.1371/journal.pgen.1007883)
Supplement: S3 Table — (DOC) [file pgen.1007883.s010.doc]

**Table S3. Primers used in this study**

| **Name** | **Sequence** |
| --- | --- |
| Circ1 | GCCGTGCTAGAGAGGCGCTA |
| Circ2 | AGCAGAATCAGATGCACGCCATTCG |
| In1 | GATAGTCGCGCACGCCATACTTC |
| In2 | CTGCATCTTCCCGGAAGGCAAG |
| P1 | TCACTGGCCGTCCTCCGG |
| P2 | AGCTATTCGTCAGGCATCCTGAAGC |
| P3 | CGCAACGAACTCACGGAGATCCTC |
| P4 | TCGTGTGTTCTCCTGAACGAAGAGGATTG |
| P5 | GCTGACGTCGATACAACCGCTCC |
| P6 | ATCGGCTCACGTCGTACACGAG |
| P7 | GGCGACATACACGAGCACGTTCTT |
| P8 | AGACCTGCGACCAGAACGATCC |
| P9 | GTTACTTGATGTAGCTTGCACGGTA |
| P10 | GGCGAAATCGCATAGATGAGTGA |
| P11 | AGTCCGTTGGTGAAGCTAGC |
| P12 | CAAAGCCTGAATGCCATATTGGG |
| P13 | GCGATTGCGTGTGTCGATGC |
| kan1 | CAGCGCATCGCCTTCTATCG |
| β2-F | TTTGCACATGCCAAGGTGTTCGAAG |
| β2-R | AGCTCACTAACGTCGTACTCGTTCG­­ |
| β3-F | AAAGGTTCAACGCAGGAAACCGT |
| β3-R | CAACCTCTGCTCAACCGACTGATC |
| β4-F | CACTTCCGCCATTGGTTACCAGC |
| β4-R | CCCAGTGCAACCAATACACCGTTT |
| β5-F | GCAGGTCATCAACAACTGAAATAGCC |
| β5-R | ATCTTCGGGCTGATATAGGCGACG |
|  |  |
|  |  |
|  |  |
|  |  |
|  |  |
|  |  |
|  |  |
